# High expression of DEC2 distinguishes chondroblastic osteosarcoma and promotes tumour growth by activating the VEGFC/VEGFR2 signalling pathway

**DOI:** 10.1111/jcmm.18462

**Published:** 2024-06-07

**Authors:** Maimaitiaili Tuerxun, Xu Zheng, Jun Xu, Quanjun Yang, Ting Yuan, Changqing Zhang, Shumin Zhou

**Affiliations:** ^1^ Department of Orthopaedic Surgery Shanghai Sixth People's Hospital Affiliated to Shanghai Jiao Tong University School of Medicine Shanghai China; ^2^ Department of Pharmacy Shanghai Sixth People's Hospital Affiliated to Shanghai Jiao Tong University School of Medicine Shanghai China; ^3^ Institute of Microsurgery on Extremities, Shanghai Sixth People's Hospital Affiliated to Shanghai Jiao Tong University School of Medicine Shanghai China

**Keywords:** Chondroblastic osteosarcoma, DEC2, phosphorylation, VEGF signalling

## Abstract

Osteosarcoma (OS) is the most common primary malignant bone tumour in children and young adults. Account for 80% of all OS cases, conventional OS are characterized by the presence of osteoblastic, chondroblastic and fibroblastic cell types. Despite this heterogeneity, therapeutic treatment and prognosis of OS are essentially the same for all OS subtypes. Here, we report that DEC2, a transcriptional repressor, is expressed at higher levels in chondroblastic OS compared with osteoblastic OS. This difference suggests that DEC2 is disproportionately involved in the progression of chondroblastic OS, and thus, DEC2 may represent a possible molecular target for treating this type of OS. In the human chondroblastic‐like OS cell line MNNG/HOS, we found that overexpression of DEC2 affects the proliferation of the cells by activating the VEGFC/VEGFR2 signalling pathway. Enhanced expression of DEC2 increased VEGFR2 expression, as well as increased the phosphorylation levels at sites Y951 and Y1175 of VEGFR2. On the one hand, activation of VEGFR2_Y1175_ enhanced cell proliferation through VEGFR2_Y1175_‐PLCγ1‐PKC‐SPHK‐MEK‐ERK signalling. On the other hand, activation of VEGFR2_Y951_ decreased mitochondria‐dependent apoptosis rate through VEGFR2_Y951_‐VARP‐PI3K‐AKT signalling. Activation of these two signalling pathways resulted in enhanced progression of chondroblastic OS. In conclusion, DEC2 plays a pivotal role in cell proliferation and apoptosis‐resistance in chondroblastic OS via the VEGFC/VEGFR2 signalling pathway. These findings lay the groundwork for developing focused treatments that target specific types of OS.

## 1 INTRODUCTION

Osteosarcoma (OS) is the most common primary bone malignant tumour in children and young adults, causing thousands of disabilities and deaths annually.^1^ OS develops from osteoblasts and cells that form the osteoid matrix.^2^ Contrary to popular opinion, OS is quite diverse, comprising several subtypes according to the predominant type of matrix within the primary tumour. Anatomically, OS presents as either intramedullary or surface lesions.^3^ Intramedullary OS can be categorized into conventional, telangiectatic, small cell, or low‐grade subtypes. About 80% of all intramedullary OS are conventional OS, which can be further subdivided into osteoblastic, chondroblastic and fibroblastic subtypes.^4^

Although various subtypes of OS exist, the clinical treatment of all OS is typically similar.^5^ The gold‐standard treatment for most OS patients is neoadjuvant chemotherapy followed by liberal surgical resection and additional adjuvant chemotherapy for high‐grade lesions.^6^ Nonetheless, the overall survival rate of OS patients has barely improved over the past decades.^7^ One reason for this stable, low survival rate may be because the standard treatment is suboptimal for certain OS subtypes. Another contributing factor may be that systemic chemotherapy comes with severe side effects, such as chemotherapy‐induced nausea and vomiting, anaemia, cardiotoxicity, nephrotoxicity and neurotoxicity, limiting benefit for patients of any subtypes.^8^

To enhance survival rate for OS patients, attempts have been made to develop specific therapies that uniquely target each OS subtype. However, those therapies achieved limited success due to the lack of OS‐specific biomarkers.^9^ This is reflected in the relative paucity of published case reports describing unique treatments for specific types of OS. For example, in Mamachan et al,^10^ a case of chondroblastic OS in the anterior maxilla was treated only by complete resection. In another case, small cell OS was treated by surgical resection followed by postoperative adjuvant chemo‐ and radio‐therapy.^11^ It is reasonable to suppose, therefore, that the discovery of OS‐specific biomarkers would help guide treatment of different subtypes of OS.

While the surface OS‐biomarkers approach to treatment has produced mixed results, attempts to identify aberrant activation of transcription factors has attracted much attention, and some progress has been made.^12^ By analysing single‐cell RNA sequencing (scRNA‐seq) data of 11 patients with osteoblastic OS and chondroblastic OS, we discovered that chondroblastic OS highly expresses the transcription factor DEC2, but osteoblastic OS does not. Differentiated embryonic chondrocytes expressed gene 2 (DEC2), also known as BHLHE41/BHLHB3/SHARP1,^13^ is one of the most important transcription factors in physiological and pathological conditions and has been studied in various tumours.^14^ Hu et al,^15^ for example, reported that DEC2 facilitates HIF‐1α stabilization, promotes HIF‐1 activation in OS, and contributes to the progression and metastasis of OS. However, the biological function(s) of DEC2 in tumours remains controversial. For example, DEC2 has suppressive effects on breast carcinoma, lung, pancreatic, and gastric cancers,^16^, ^17^, ^18^, ^19^ but some evidence suggests that it contributes to the progression of endometrial carcinomas, oesophageal cancer, breast cancers, and osteosarcoma.^15^, ^20^, ^21^, ^22^ However, research on DEC2 functioning in tumours is just beginning, and detailed mechanistic information and pathway involvement is still lacking. Detailed mechanistic studies would greatly advance our understanding of what role DEC2 plays in the progression of chondroblastic OS, which could improve treatment and prognosis of chondroblastic OS. Therefore, we hypothesize that DEC2 plays a pivotal role in the progression of chondroblastic OS through activating the hypoxia signalling pathway.

In this study, we demonstrated that DEC2 expression is significantly increased in chondroblastic OS clinical samples, as well as in a chondroblastic‐like OS cell line in vitro. Moreover, we found that DEC2 contributes to enhanced chondroblastic‐like OS cell proliferation and to apoptosis‐resistance by regulating phosphorylation of VEGFR2. High expression of DEC2 contributes to enhanced chondroblastic OS cell proliferation through VEGFR2_Y1175_‐PLCγ1‐PKC‐SPHK‐MEK–ERK signalling and decreases the apoptosis rate through VEGFR2_Y951_‐VARP‐PI3K‐AKT signalling. Together, this results in the progression of chondroblastic OS. Thus, our results suggest that DEC2 plays a pivotal role in cell proliferation and apoptosis‐resistance in chondroblastic‐like OS cells via VEGFC/VEGFR2 signalling. These findings could pave the way to developing new therapies against chondroblastic OS.

## 2 METHODS AND MATERIALS

### 2.1 Cell lines and culturing

Three human osteosarcoma (OS) cell lines MNNG/HOS, U2OS and 143B were purchased from American Type Culture Collection (ATCC, Manassas, VA, USA). Bone marrow stromal cells (BMSCs) were harvested from patients with open fractures who underwent debridement at Shanghai Jiao Tong University Affiliated Sixth People's Hospital, and written informed consent was acquired and signed by the patients. MNNG/HOS, 143B, and U2OS cells were cultured in DMEM (Corning, Corning, NY, USA); BMSCs were cultured in α‐MEM (Corning, USA). All cell lines were maintained at 37°C in a humidified cell‐culture chamber with an atmosphere of 5% CO_2_. Culture media were supplemented with 10% fetal bovine serum (Gibco, Waltham, MA, USA) and 1% Penicillin‐Streptomycin (V900929, Sigma).

### 2.2 Stable cell line construction

For stable cell line construction, HEK293T cells were co‐transfected with lentivirus packing vectors and LV shuttle plasmids containing full‐length DEC2(NM_030762.3)and siRNA against DEC2. After 48 h, the supernatant containing lentivirus was collected, purified and followed by titre determinization. Then, the lentivirus was added to the culture medium of MNNG/HOS cells at a MOI = 10.0. After 72 h, the culture medium was changed to a new medium that contained puromycin (1.0 μg/mL) to select the positive cells. Finally, stable cells overexpressing (MNNG/HOS‐D2) and knockdown DEC2 (MNNG/HOS‐siD2) were verified by RT–qPCR and WB (Figure S2A–D). All the information of the vector and sequences of full‐length DEC2 and siRNA against DEC2 has been provided in Table S2.

### 2.3 Total RNA extraction and real‐time quantitative PCR

Trizol reagent (Invitrogen, USA) was used to extract total RNA from cell lines. Reverse transcription was achieved by a Revert Aid First Strand cDNA Synthesis Kit (Invitrogen, USA). Quantitative real‐time PCR (RT‐qPCR) assays were carried out on an ABI Prism 7900HT real‐time system (Applied Biosystems) by applying specific primers and SYBR gene PCR master mix (Invitrogen, USA). The 2^−ΔΔCt^ approach was used to calculate the relative mRNA expression of different genes. All primers are shown in Table S3.

### 2.4 Western blot and reagent

RIPA solution (EpiZyme, PC102) containing proteinase inhibitor (Invitrogen, 36,978) and phosphatase inhibit were added to culture cells to gain lysis solution. Then, the lysates were centrifuged (13,000 ***g***/15 min, 4°C) and discarded precipitate. Equal amounts of collected total proteins were separated by SDS‐PAGE and transferred to a PVDF membrane. Giving the membranes a dip in 5% milk at room temperature (RT) for 1 h. Then, proteins were detected by incubated membranes in primary antibodies solution at 4°C overnight. After that, HRP‐linked anti‐IgG antibodies were employed as secondary antibodies. Primary antibodies were shown in Table S4.

### 2.5 Cell proliferation assay

Real‐time cellular analysis (RTCA) (ACEA Biosciences, USA) was used to evaluate cell proliferation.^23^ First, the baseline value was measured in 100 ul of culture medium preincubated at 37°C in a cell incubator for 1 h. Then, the cells were seeded in wells at a density of 2.0 × 10^3^ cells per well. The attachment and proliferation of cells were measured by the RTCA system for 6 and 168 h, respectively.

### 2.6 Colony formation assay

For colony formation ability detection, cells were incubated in a six‐well plate for 2 weeks at a density of 1000 cells per wells. After being fixed with 4% paraformaldehyde (PFA), the cells were stained with crystal violet for half an hour. Colonies that contain over 50 cells were recorded and counted by the ImageJ software (Rasband, W.S., ImageJ, U. S. National Institutes of Health, Bethesda, Maryland, USA, http://imagej.nih.gov/ij/, 1997–2015).

### 2.7 Cell migration and invasion assays

Cell migration and invasion assays were performed in 24‐well plates with 8‐mm pore size chamber inserts (Corning, NY, USA), following established procedures. For migration assays, 4 × 10^4^ cells were placed into each well of the upper chamber with the non‐coated membrane. For invasion assays, 4 × 10^4^ cells were placed into the upper chamber with the Matrigel‐coated membrane, which was diluted with serum‐free culture medium. In both assays, cells were suspended in 200 μL of DMEM without fetal bovine serum when they were seeded into the upper chamber. In the lower chamber, 800 μL of DMEM supplemented with 10% fetal bovine serum was added. Cells that moved to the bottom surface of the chamber were fixed with 100% methanol for 20 min and stained with 0.1% crystal violet for 30 min. Then imaged and the cell numbers counted under microscope.

### 2.8 Cell apoptosis analysis

Cells were cultured in a six‐well plate, cells were washed with a cold Phosphate‐Buffered Saline (PBS) solution followed by digested with trypsin. Digested cells were processed by the Annexin V‐PI kit (Beyotime, C1062L) according to the manufacturer's instructions. The result was measured by flow cytometry.

### 2.9 RNA‐seq and analysis

Total RNA was extracted from MNNG/HOS‐siD2, MNNG/HOS‐D2, and control cell lines by Trizol reagent (Invitrogen, USA). The RNA‐Seq libraries were synthesized by the TruSeq™ RNA Sample Preparation Kit (Illumina, USA) following the standard Illumina guidelines. After purification, the quantification and validation of the libraries were performed by Qubit® 2.0 Fluorometer (Life Technologies, USA) and Agilent 2100 bioanalyzer (Agilent Technologies, USA), and finally, the library was sequenced by Illumina NovaSeq 6000 (Illumina, USA). The library construction and sequencing were performed by Sinotech Genomics Co., Ltd (Shanghai, China). The selection criteria of differential expressed genes (DEG) was a less than five percentage false discovery rate (FDR) and changed expression higher than 1.5 or lower than 0.67‐fold. All cell lines were tested three times. The raw RNA‐seq data were uploaded to NCBI SRA database. The SRA accession number: PRJNA931766.

### 2.10 Subcutaneous tumour model

Female nude mice ranging from 4 to 6 weeks were purchased from the Laboratory Animal Research Center of Shanghai Sixth People's Hospital, and all operations were approved by the Animal Research Committee of Shanghai Sixth People's Hospital. After anaesthesia by pentobarbital sodium, 100 μL of cell suspension containing 1 × 10^6^ cells were injected into the nude mouse flank.^24^ Tumours were measured by researchers until the longest diameter of the largest tumour reached 200 mm.

### 2.11 Immunofluorescence (IHC) analysis

Cells were cultured in coverslips which were placed in a 24‐well plate. When confluent up to 30%, cells were fixed with 4% PFA for 20 min at RT. After being washed with PBS, fixed cells were blocked by incubating in QuickBlock™ (Beyotime, P0260) for 1 h. Then, cells were washed by PBS three times and incubated with rabbit anti‐ BHLHB3 (1:200, AF0442, Affinity) and mouse anti VEGFR2 (1:200, Sc‐6251, SANTA CRUZ) overnight at 4°C. Subsequently, discarded primary antibody and washed by PBS three times, cells were incubated with Alexa Fluor 488‐conjugated goat anti‐rabbit antibody and Alexa Fluor 647‐conjugated goat anti‐mouse antibody at RT for 1 h. DAPI was used to stain nuclei. Images were taken by Confocal Microscope (Leica Microsystems).

### 2.12 Immunohistochemical analysis

The OS tissues excised from the subcutaneous tumour model were embedded in paraffin and then cut into 4 μm sections and deparaffinized. The sections were blocked with 5% bovine serum albumin (BSA) at 37°C for 30 min. After that, specific primary antibodies were added to the samples and incubated overnight at 4°C. Then, the cells were washed three times with PBS and incubated with HRP‐linked anti‐IgG at 37°C for 30 min. Washing by PBS again and stained in 3,3′‐diaminobenzidine (DAB) for 10 min. Finally, the samples were counterstained, dehydrated, covered with cover glass and photographed with DM6B (Leica, BRD). The mean density of staining, calculated using Image Pro‐Plus (Media Cybernetics, Inc., Rockville, MD, USA) as previously described,^25^ was used to quantify the staining.

### 2.13 Statistical analyses

The data were analysed by SPSS 25.0 software and presented as the mean ± SD. The differences between experimental and control groups were analysed by two‐tailed Student's *t* test. ns means *p* > 0.05, *p < 0.05, **p < 0.01, and ***p < 0.001.

## 3 RESULTS

### 3.1 DEC2 is expressed at higher levels in chondroblastic OS compared with osteoblastic OS

Before examining possible differences in gene expression, we first mined sequencing data from our previous study^26^ of tumour cells in 11 OS patients (8 with osteoblastic OS and 3 with chondroblastic OS). Of the osteoblastic OS cases, six were primary lesions, one was a recurrent lesion and one was a lung metastatic lesion. Of the chondroblastic OS cases, one was a primary lesion, one was a recurrent lesion and one was a lung metastatic lesion.^26^ All 11 patients in that study had undergone preoperative chemotherapy.^26^ In all three chondroblastic OS cases, necrosis rates were below 90%; in 2 of 8 osteoblastic OS cases, necrosis rates were above 90% (Table S1).

**FIGURE 1:**
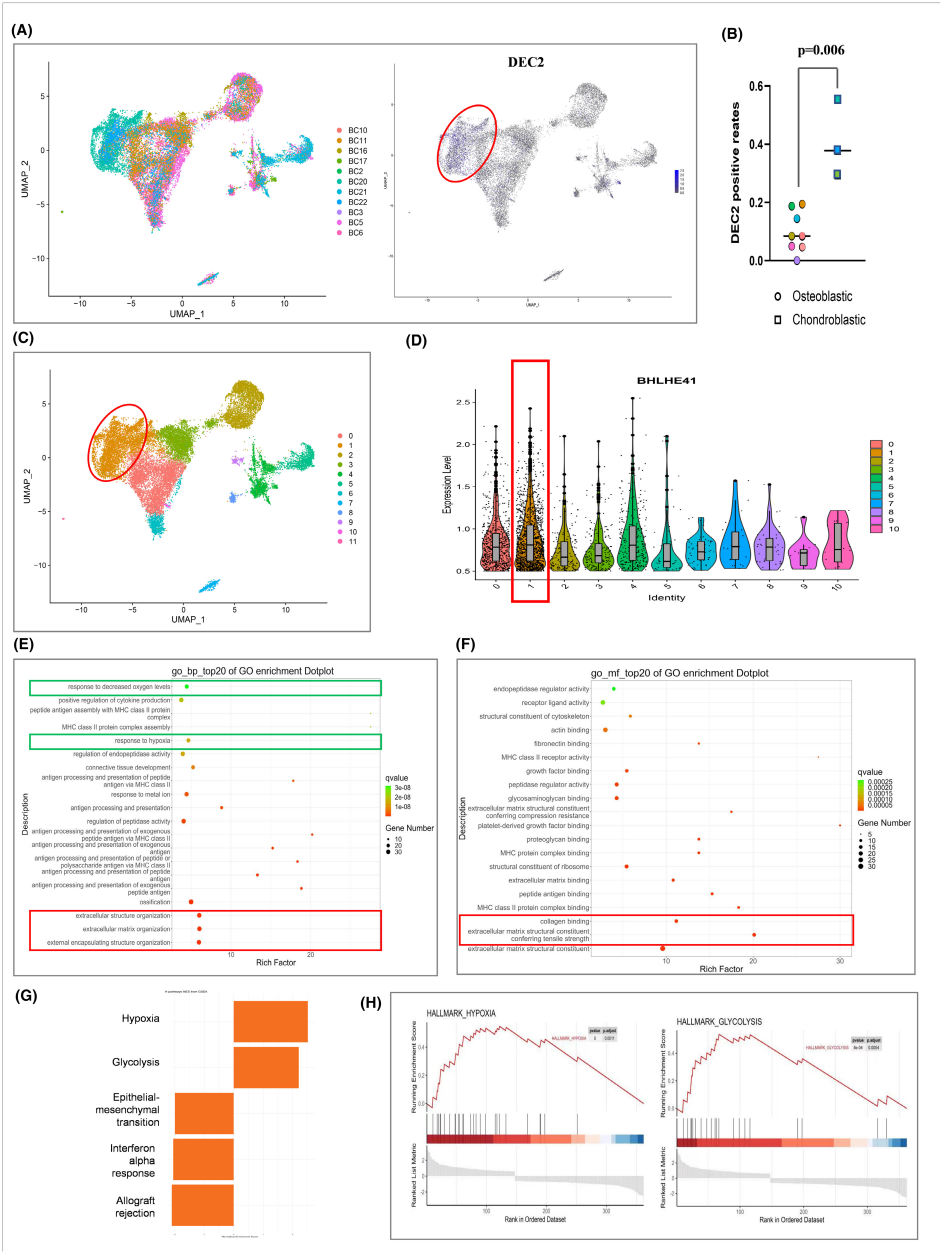
DEC2 at the single cell level is more highly expressed in chondroblastic OS compared to osteoblastic OS. (A) Cell atlas of DEC2 expression in OS clinical samples shown in UMAP plots. (B) DEC2 positive rates in chondroblastic and osteoblastic OS samples. (C) Marker genes used to annotate cell types and cell atlas of different cell clusters. Red oval superimposed on plots shows cluster 1 in which DEC2 was predominately expressed. (D) Violin plot comparing expression levels of DEC2 in the 12 cell clusters. (E, F) KEGG pathway enrichment analysis showing top 20 GO enrichments in cell cluster 1. (G) Gene set enrichment analysis was performed for positively or negatively enriched pathways in cell cluster 1. (H) Hypoxia pathway and glycolysis pathway are the two positively enriched pathways.

Next, to identify transcription factors that might be aberrantly expressed in osteoblastic and chondroblastic OS, we first screened for transcription factors that were previously reported by other OS studies to be overexpressed.^27^ We considered expression levels >0.5 to be positive for overexpression. We explored two datasets, GSE152048 and GSE162454.^28^, ^29^, ^30^ First, we conducted cluster analysis on osteosarcoma (OS) cells using the single‐cell RNA‐seq data from GSE152048, and investigated the expression patterns of DEC2 on OS cells. Our findings delineate distinct expression profiles of DEC2 within the tumour micro‐environment. DEC2 was mainly expressed in chondroblastic OS, with relatively little in osteoblastic OS (Figure 1A,B). Uniform Manifold Approximation and Projection (UMAP) analysis revealed 12 different cell clusters (Figure 1C). DEC2 was predominately expressed in cluster 1 (Figure 1D). KEGG pathway enrichment analysis revealed that the top 20 GO enrichments in cell cluster 1 belonged to response of oxygen levels and extracellular matrix organization pathways (Figure 1E,F, Figure S1A). We also expanded our analysis to integrate single‐cell RNA‐seq data from GSE162454. Our examination revealed that DEC2 is primarily expressed in tissue stem cells and certain osteosarcoma subtypes.

Gene set enrichment analysis showed two positively (Figure 1G,H) and three negatively enriched pathways (Figure S1B) in cluster 1; the hypoxia pathway and glycolysis pathway were the main positively enriched pathways. These two pathways are related to oxygen metabolism. These results characterizing cell cluster 1 are capable of producing extracellular matrix and are suitable to hypoxic conditions, both of which are consistent with the characteristics and requirements of chondroblastic cells.^31^

### 3.2 DEC2 positively regulates the growth of chondroblastic‐like OS cells

**FIGURE 2:**
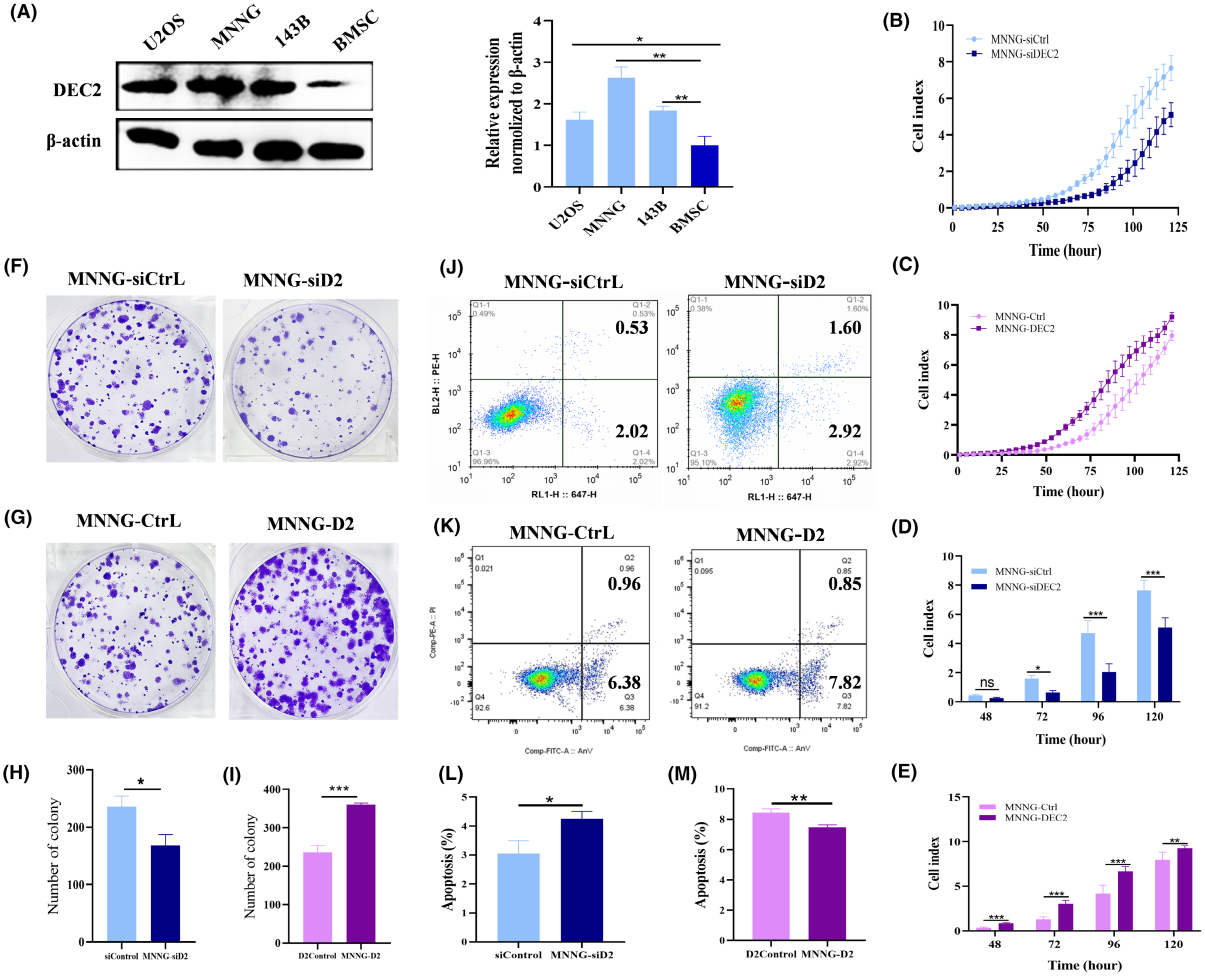
DEC2 positively regulates growth in chondroblastic‐like OS cell line. (A) Western blot of DEC2 protein expression levels in OS cells and bone marrow mesenchymal stromal cells (BMSC) (control). Quantitation of relative protein expression. (B–E) Results of RTCA of MNNG/HOS‐DEC2, MNNG/HOS‐siDEC2, and their respective control cells. (F–I) Representative images of colony formation assay results for MNNG/HOS‐DEC2, MNNG/HOS‐siDEC2, and their respective control cells. (J–M) Flow cytometry analysis of cell apoptosis (Annexin V/PI staining) rates of MNNG/HOS‐DEC2, MNNG/HOS‐siDEC2, and their respective control cells. Data expressed as means ± SD (*N* = 3) for experimental results. *p < 0.05; **p < 0.01; ***p < 0.001 for all panels.

To date, there are no well‐accepted human OS cell lines that are recognized as being of the chondroblastic subtype. Although Sato et al. established a novel osteosarcoma cell line, referred to as CHOS, which was derived from primary chondroblastic osteosarcoma from a 58‐year‐old man, but it is not commercialized.^32^ So as an alternative, we employed commonly used human OS cell lines (MNNG/HOS, U2OS, 143B) for our DEC2 expression screening analyses. Western blot analysis for DEC2 proteins revealed that DEC2 was most highly expressed in MNNG/HOS cells (Figure 2A). Thus, we hypothesized that MNNG/HOS cells are like chondroblastic OS cells. Thus, throughout this report we use the term ‘chondroblastic‐like OS cells’ when referring to MNNG/HOS cells.

In order to determine possible roles DEC2 plays in chondroblastic‐like OS cells, we constructed two DEC2‐regulated stable cell lines based on MNNG/HOS cells: MNNG/HOS‐DEC2 (in which DEC2 is overexpressed) and MNNG/HOS‐siDEC2 (in which DEC2 is knocked down). These two lines, as well as their respective control cell lines, were constructed using a lentivirus system; their DEC2 expression, or lack of expression, was verified at both mRNA and protein levels. The ratios of DEC2 mRNA expression to protein expression were as follows: DEC2‐overexpressing cells, 85.41 ± 16.67 to 1.82 ± 0.53 (MNNG/HOS‐DEC2 to DEC2 control, respectively); DEC2‐knockdown cells, 0.31 ± 0.08 vs. 0.55 ± 0.24 (MNNG/HOS‐siD2 to siD2 control, respectively) (Figure S2A,B).

To examine whether the altered expression of DEC2 affects cell function, we first assessed OS cell proliferation by Real Time Cellular Analysis (RTCA). At the indicated times, RTCA showed that cell proliferation was significantly increased in MNNG/HOS‐DEC2 cells (ratio of MNNG/HOS‐DEC2 to DEC2 control, 2.53 ± 0.62, 2.48 ± 0.74, 1.68 ± 0.45, and 1.18 ± 0.17 at 48, 72, 96, and 120 h, respectively) (Figure 2C,E). By contrast, cell proliferation in MNNG/HOS‐siDEC2 cells was slightly decreased compared to control cells (ratio of MNNG/HOS‐siDEC2 to siDEC2 control, 0.55 ± 0.05, 0.40 ± 0.18, 0.43 ± 0.72, and 0.67 ± 0.66 at 48, 72, 96 and 120 h, respectively) (Figure 2B,D). We confirmed a similar pattern in colony formation assays. Compared with that of controls, colony formation of HOS/MNNG‐DEC2 and HOS/MNNG‐siDEC2 cells, respectively, increased or decreased (ratio of HOS/MNNG‐DEC2 to DEC2 control and ratio of MNNG/HOS‐siDEC2 to siD2Control were 1.53 ± 0.13 and 0.71 ± 0.03, respectively) (Figure 2F–I).

We also measured cell apoptosis in MNNG/HOS‐DEC2, MNNG‐siDEC2 cells, and their respective control cells by flow cytometry. We observed enhanced apoptosis in the MNNG/HOS‐siDEC2 group (4.52% vs. 2.55%) and attenuated apoptosis in the MNNG/HOS‐DEC2 group (7.34% vs. 8.67%) compared with control cells (Figure 2J–M).

Cell migration and invasion of MNNG/HOS‐DEC2, MNNG/HOS‐siDEC2 and their respective control cells were assessed with transwell assay (Figure S2C–F). Slight change was observed among the groups.

These results demonstrated that the expression of DEC2 was positively correlated with cell proliferation, colony formation and apoptosis‐resistance in chondroblastic‐like OS cells.

### 3.3 Upregulation of DEC2 activates VEGF signalling pathway in chondroblastic‐like OS cells

**FIGURE 3:**
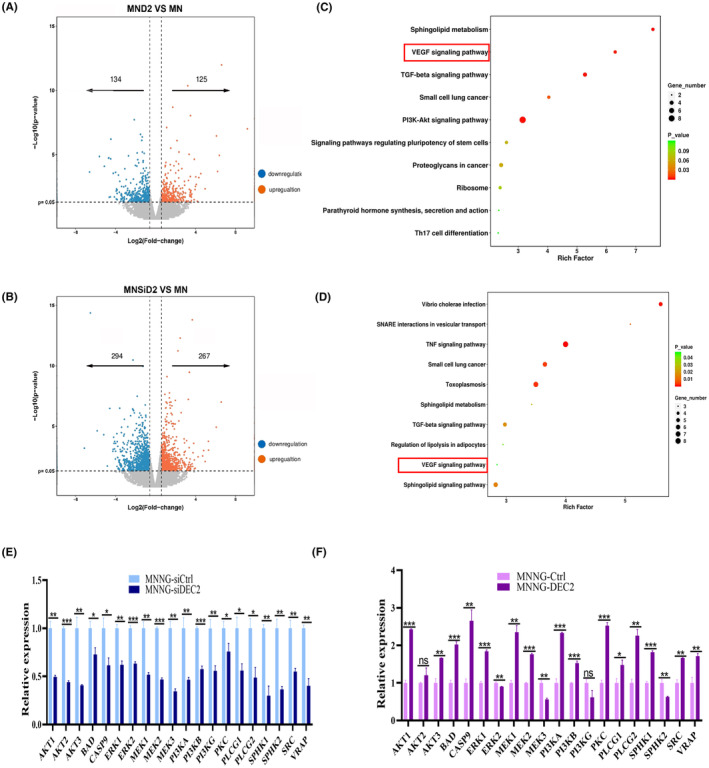
Upregulation of DEC2 activates the VEGF signalling pathway in chondroblastic‐like OS cells. (A, B) Volcano plots showing differentially expressed genes (DEGs) in DEC2‐overexpressing MNNG/HOS‐DEC2 cells (panel A) and DEC2‐knockdown MNNG/HOS‐siDEC2 cells (panel B) relative to their respective control cells, as identified by transcriptome RNA‐seq analysis. (C, D) KEGG pathway enrichment analysis of DEGs identified in DEC2‐overexpressing MNNG/HOS‐DEC2 cells (panel C) and DEC2‐knockdown MNNG/HOS‐siDEC2 cells (panel D) relative to their respective control cells. (E, F) Among the DEGs, genes that are highly related to the VEGF pathway were verified by RT‐qPCR to be expressed in the DEC2‐altered cells and their controls, means ± SD (*N* = 3). *P < 0.05; **P < 0.01; ***P < 0.001; ns, not significant.

To determine the possible mechanisms underlying DEC2's functions in chondroblastic‐like OS cells, we performed transcriptome sequencing (RNA‐seq) analysis on MNNG/HOS‐DEC2, MNNG/HOS‐siDEC2, and their respective control cells using cutoff values of >1.5 or <0.67. In DEC2‐overexpressing cells, 259 genes were differentially expressed compared with the control. By contrast, in DEC2‐knockdown cells, 561 genes were differentially expressed compared with the control. These differentially expressed genes (DEGs) are shown in Figure 3A,B.

Next, we did Kyoto Encyclopedia of Genes and Genomes (KEGG) analysis on these DEGs. Genes related to the VEGF signalling pathway were differentially expressed in both of the DEC2‐engineered OS cell lines (MNNG/HOS‐DEC2, MNNG/HOS‐siDEC2) as compared to their respective control cells (Figure 3C,D). We also verified the KEGG analyses via RT‐qPCR by assessing the expression of genes related to VEGF signalling pathways that were identified during transcriptome sequencing and literature mining. RT‐qPCR performed on MNNG/HOS‐DEC2, MNNG/HOS‐siDEC2, and their respective control cells revealed that expression of genes related to VEGF signalling were indeed differentially expressed (Figure 3E,F). DEC2 upregulation in MNNG/HOS‐DEC2 cells resulted in the activation of VEGF signalling pathways, whereas downregulation or knockdown of DEC2 in MNNG/HOS‐siDEC2 cells inhibited the activation of VEGF signalling pathways.

### 3.4 DEC2 activates VEGF signalling by increasing expression and phosphorylation of VEGFR2 in chondroblastic‐like OS cells

**FIGURE 4:**
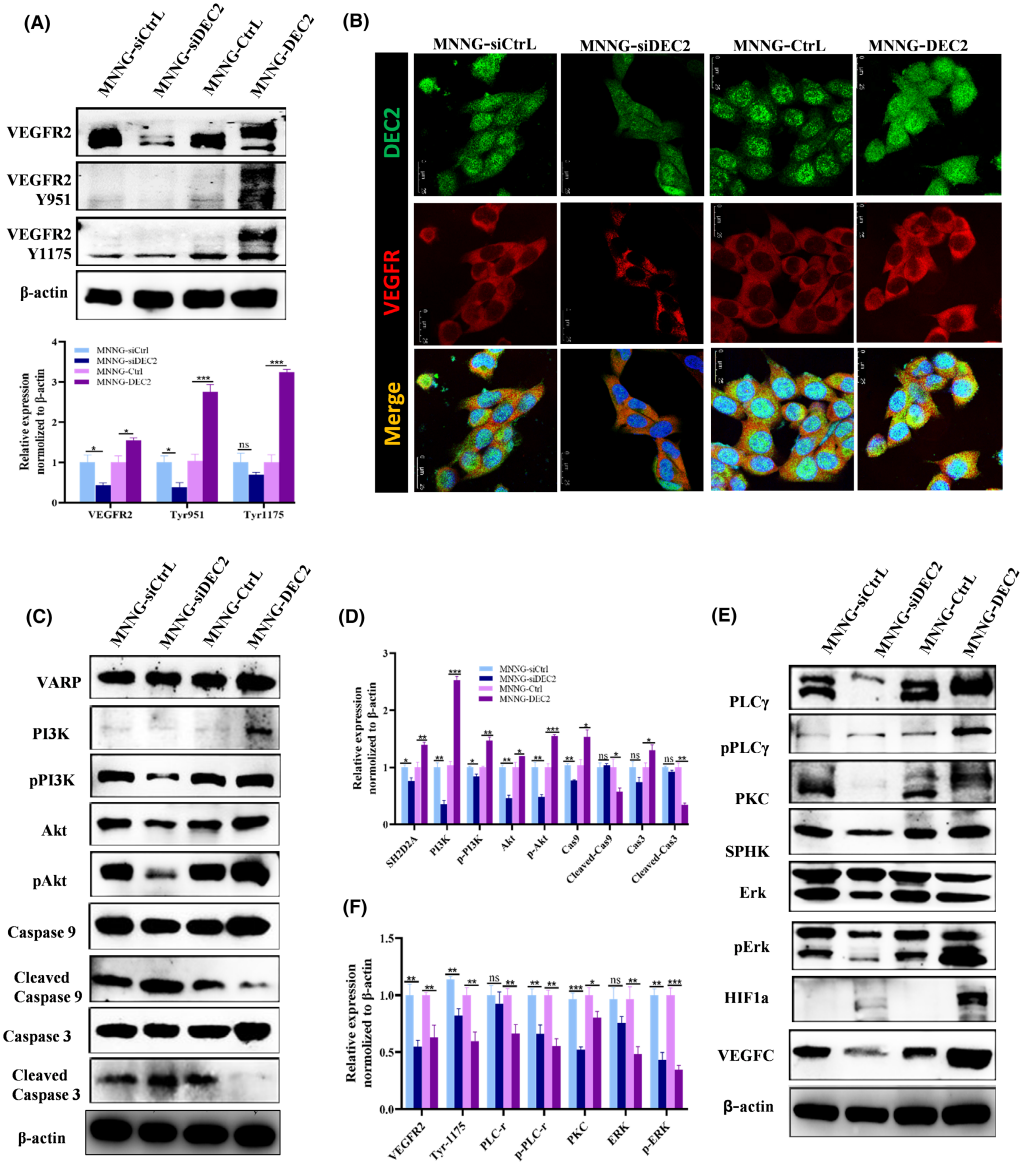
DEC2 activates VEGF signalling by increasing the expression and phosphorylation of VEGFR2 in chondroblastic‐like OS cells. (A) Western blot showing levels of VEGFR2 protein expression and phosphorylation in DEC2‐overexpressing MNNG/HOS‐DEC2 cells and DEC2‐knockdown MNNG/HOS‐siDEC2 cells. (B) Representative immunofluorescent images of MNNG/HOS‐DEC2 cells, MNNG/HOS‐siDEC2 cells, and their respective control cells stained for VEGFR2 and DEC2. (C, D) Western blot showing expression levels of proteins of the VARP‐PI3K‐AKT‐Caspase3 signalling axis in MNNG/HOS‐DEC2 cells, MNNG/HOSsiDEC2 cells, and their respective control cells. Relative expression was normalized to β‐actin expression. (E, F) Western blot showing expression levels of proteins of the PLCγ1‐PKC‐SPHK‐MEK–ERK signalling axis in MNNG/HOS‐DEC2 cells, MNNG/HOS‐siDEC2 cells, and their respective control cells. For panels A, D, and F, quantitation of relative protein expression levels is presented as means ± SD (*N* = 3); *p < 0.05; **p < 0.01; ***p < 0.001; ns, not significant.

To verify that DEC2 activates VEGF signalling pathways, we measured the expression of VEGFR2 protein and the phosphorylation of two of its tyrosine sites (Y951 and Y1175). As the main receptor in VEGF signalling, VEGFR2 participates in cell proliferation and apoptosis.^33^ Western blot analysis showed that VEGFR2 expression was elevated in MNNG/HOS‐DEC2 cells but greatly reduced in MNNG/HOS‐siDEC2 cells (Figure 4A). Double immunofluorescence staining against DEC2 and VEGFR2 of MNNG/HOS‐DEC2 cells and MNNG/HOS‐siDEC2 cells were consistent with these results (Figure 4B). DEC2 overexpression also significantly increased the phosphorylation of VEGFR2_Y1175_ and VEGFR2_Y951_ (Figure 4A).

To determine how phosphorylation of these two sites affects proteins downstream of VEGFR2_Y1175_ and VEGFR2_Y951_, we assessed the expression of these downstream proteins by Western blotting. As shown in Figure 4C, the activation of VEGFR2_Y951_ also resulted in the activation of the VARP‐PI3K‐AKT‐Caspase9‐Caspase3 signalling pathway in MNNG/HOS‐DEC2 cells. The ratios of MNNG/HOS‐DEC2 to DEC2 control cells were as follows: VARP, 1.40 ± 0.11; p‐PI3K, 1.47 ± 0.04; p‐AKT, 1.55 ± 0.11; cl‐Caspase9, 0.58 ± 0.13; cl‐Caspase3, 0.34 ± 0.05 (Figure 4D). By contrast, the activation of VEGFR2_Y1175_ resulted in activation of the PLCγ1‐PKC‐SPHK‐MEK–ERK‐HIF1a‐VEGFC signalling pathway in MNNG/HOS‐DEC2 cells (Figure 4E). The ratios of MNNG/HOS‐DEC2 to DEC2Control were as follows: p‐PLCγ1, 1.39 ± 0.08; PKC, 1.45 ± 0.14; SPHK,1.63 ± 0.09; p‐ERK,1.78 ± 0.02; HIF‐1α,3.16 ± 0.11; VEGFC, 2.83 ± 0.37 (Figure 4F). In MNNG/HOS‐siDEC2 cells, the expression pattern of these proteins were opposite of that observed in MNNG/HOS‐DEC2 cells (Figure 4C–F).

### 3.5 VEGFR2 inhibition reduces DEC2‐enhanced chondroblastic‐like OS cell proliferation

**FIGURE 5:**
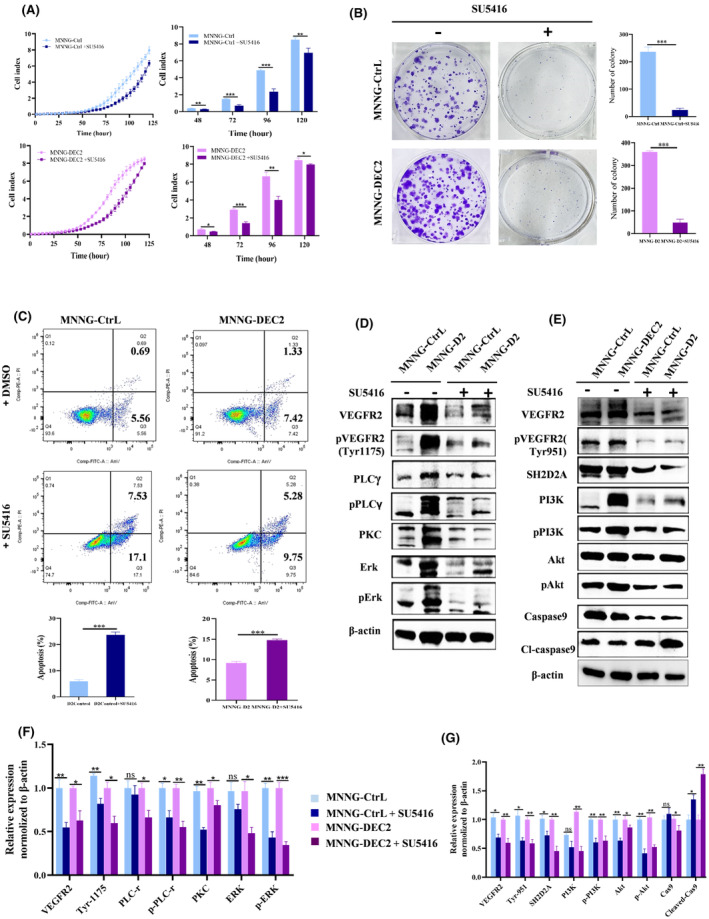
VEGFR2 inhibition reduces DEC2‐enhanced chondroblastic‐like OS cell proliferation. (A) Proliferation of MNNG/HOS‐DEC2 cells (overexpressing DEC2) and DEC2 control cells (MNNG/HOS‐Ctrl) in culture after adding 40 μm/mL of SU5416 (VEGFR2 inhibitor) or DMSO with equal volume (control) over 5 days (left), and daily average (right). Cell index is a dimensionless parameter that reflects the proportion of cells attached in a cell culture monolayer. Cell index is zero when cells are not present or do not adhere to the plate surface; cell index increases progressively and proportionally as cells become attached to the monolayer. (B) Representative brightfield images of MNNG/HOS‐DEC2 and DEC2 (MNNG/HOS‐Ctrl) control cells treated with either 7.5 μM SU5416 or DMSO. Mean number cell colonies after 2 weeks of culturing. The cell cultures were stained with crystal violet. (C) Flow cytometric analysis (Annexin V/PI staining) of cell apoptosis rates of MNNG/HOS‐DEC2 and DEC2 (MNNG/HOS‐Ctrl) control cells after 40 μM SU5416 or DMSO treatment for 48 h. Mean percentage apoptosis from flow analysis (bottom). (D, F) Western blots showing the expression levels of proteins of PLCγ1‐PKC‐SPHK‐MEK‐ERK signalling pathway in MNNG/HOS‐DEC2 and DEC2 (MNNG/HOS‐Ctrl) control cells after SU5416 (40 μM) or DMSO treatment for 48 h. Quantitation of relative expression of proteins (normalized to β‐actin). (E, G) Western blots showing expression levels of proteins of VARP‐PI3K‐AKT‐Caspase 3 signalling pathway in MNNG/HOS‐DEC2 and DEC2 control (MNNG/HOS‐Ctrl) cells after SU5416 (40 μM) or DMSO treatment for 48 h. Quantitation of relative expression of proteins. Mean ± SD, *N* = 3 for all experiments. *p < 0.05; **p < 0.01; ***p < 0.001 for all panels.

To bolster our results on VEGFR2 involvement in chondroblastic‐like OS growth, we treated MNNG/HOS‐DEC2 and DEC2 control cells with the VEGFR2 inhibitor (SU5416, MedChemExpress, NJ, USA) at a concentration of 40 μm/mL or DMSO with equal volume (control) in vitro for 48 h. SU5416 reduced cell proliferation, confirming that VEGFR2 is involved in MNNG/HOS‐DEC2 cell proliferation. Time‐specific ratios of MNNG/HOS‐DEC2 + SU5416 to MNNG/HOS‐DEC2 + DMSO were: 48 h, 0.65 ± 0.04; 72 h, 0.49 ± 0.06; 96 h, 0.60 ± 0.08; 120 h, 0.94 ± 0.03 (Figure 5A). The time‐specific ratios of DEC2 control+SU5416 to DEC2 control + DMSO were: 48 h, 0.63 ± 0.14; 72 h, 0.45 ± 0.06; 96 h, 0.46 ± 0.08; 120 h, 0.79 ± 0.07 (Figure 5A).

Colony formation assays assessing VEGFR2 inhibition produced similar results as the RTCA experiment. Colony numbers were dramatically reduced in MNNG/HOS‐DEC2 cells and control cells when treated with SU5416 compared to colony numbers after DMSO treatment. The ratio of MNNG/HOS‐DEC2+SU5416 to MNNG/HOS‐DEC2+DMSO and the ratio of DEC2 control+SU5416 to DEC2 control + DMSO were 0.13 ± 0.04 and 0.11 ± 0.03, respectively (Figure 5B).

Apoptosis rates were also affected by VEGFR2 inhibition, as shown by flow cytometric analysis (Annexin V/PI staining) (Figure 5C). After SU5416 treatment, apoptosis was significantly enhanced in MNNG/HOS‐DEC2 cells (15.03% vs. 8.75%) and DEC2 control cells (24.63% vs. 6.25%), as compared to their DMSO‐treated counterparts (Figure 5C).

Western blot analysis showed that expression levels of VEGF signalling pathway‐related proteins in MNNG/HOS‐DEC2 and DEC2 control cells were significantly decreased after SU5416 treatment, and the levels of apoptosis‐related proteins (e.g., cleaved‐caspase9) were significantly increased, when compared to their DMSO‐treated counterparts (Figure 5D–G). These results supported the above findings of VEGFR2 involvement in chondroblastic‐like OS cell proliferation and apoptosis‐resistance.

### 3.6 VEGFC rescues the decreased proliferation of DEC2‐knockdown chondroblastic‐like OS cells

**FIGURE 6:**
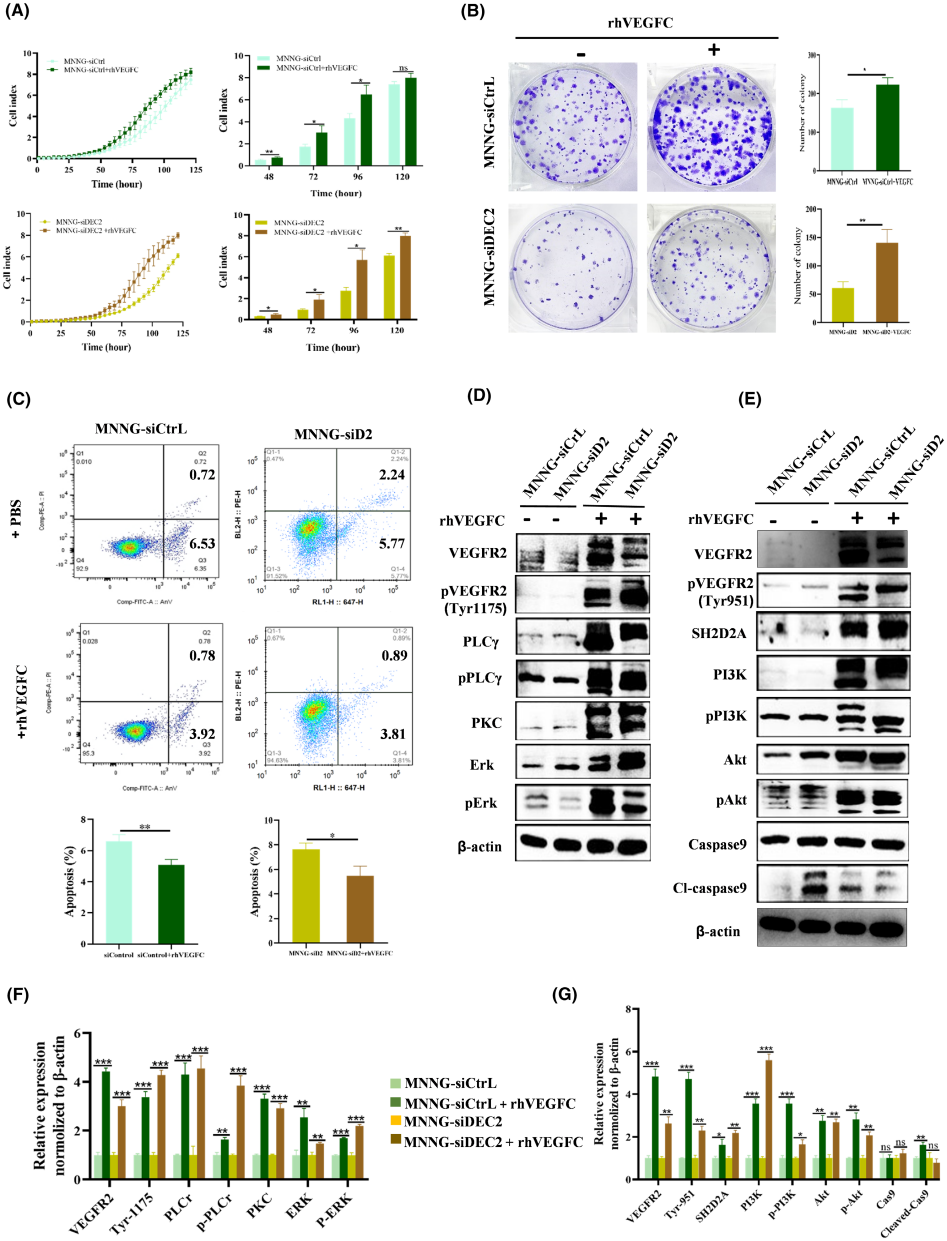
VEGFC rescued the decreased proliferation of DEC2‐knockdown chondroblastic‐like OS cells. (A) Results of RTCA of MNNG/HOS‐siDEC2 and siDEC2Control cells after 200 nM/mL VEGFC or PBS treatment with equal volume (control) for 48 h. (B) Representative brightfield images of MNNG/HOS‐siDEC2 and siDEC2control cells after treatment with either 50 nM/mL VEGFC or PBS in the colony formation assay. The cell cultures were stained with crystal violet. (C) Flow cytometry analysis of cell apoptosis rates of MNNG/HOS‐siDEC2 and siD2Control cells after 200 nm/mL VEGFC or PBS treatment for 48 h. (D, F) Western blot showing expression levels of proteins of PLCγ1‐PKC‐SPHK‐MEK–ERK signalling pathway in MNNG/HOS‐siDEC2 and siD2Control cells after 200 nm/mL VEGFC or PBS treatment for 48 h. Quantitation of relative expression of proteins. (E, G) Western blot showing expression levels of proteins of VARP‐PI3K‐AKT‐Caspase3 signal pathway in MNNG/HOS‐siDEC2 and siD2Control cells after 200 nm/mL VEGFC or PBS treatment for 48 h. Quantitation of relative expression of proteins. Mean ± SD, *N* = 3 for all experiments. *p < 0.05; **p < 0.01; ***p < 0.001 for all panels.

To further test our hypothesis that DEC2 plays an important role in chondroblastic‐like OS cell proliferation, we conducted a rescue experiment by treating MNNG/HOS‐siDEC2 and siDEC2 control cells with 200 nm/mL VEGFC or PBS with equal volume (control) in vitro for 48 h. We surmised that the activation of VEGFC signalling could reverse the reduction in chondroblastic‐like OS cell growth caused by DEC2 knockdown. RTCA assays confirmed that cell proliferation was elevated in VEGFC‐treated MNNG/HOS‐siDEC2 and siDEC2 control cells compared to PBS‐treated MNNG/HOS‐siDEC2 and siDEC2 control cells. The ratios of MNNG/HOS‐siDEC2+VEGFC to MNNG/HOS‐siDEC2+PBS were: 48 h, 1.57 ± 0.34; 72 h, 2.01 ± 0.61; 96 h, 2.10 ± 0.55; 120 h, 1.31 ± 0.08. The ratios of siDEC2 control + VEGFC to siDEC2 control + PBS were: 48 h, 1.47 ± 0.21; 72 h, 1.80 ± 0.49; 96 h, 1.53 ± 0.28; 120 h, 1.08 ± 0.11 (Figure 6A).

Results from colony formation assays and apoptosis experiments were consistent with the results from the rescue experiments. Colony numbers increased in MNNG/HOS‐siDEC2 cells and siDEC2 control cells when treated with VEGFC compared with PBS. The ratio of MNNG/HOS‐siDEC2+VEGFC to MNNG/HOS‐siDEC2+PBS and the ratio of siDEC2control+VEGFC to siDEC2control+PBS were 2.36 ± 0.48 and 1.38 ± 0.18, respectively (Figure 6B). Next, we assessed apoptosis in MNNG/HOS‐siDEC2 and siDEC2 control cells treated with either VEGFC or PBS. Apoptosis in the VEGFC‐treated group was significantly reduced compared to their PBS‐treated counterparts (MNNG/HOS‐siDEC2, 4.7% vs. 8.01%; siDEC2 control cells, 4.7% vs. 7.07%; Figure 6C).

Western blot analysis showed that expression levels of proteins in the VEGF signalling pathway in VEGFC‐treated MNNG/HOS‐siDEC2 cells and siDEC2 control cells were significantly increased compared to their PBS‐treated control counterparts (Figure 6D–G). By contrast, expression levels of apoptosis‐related proteins (cleaved‐caspase9) were significantly decreased in VEGFC‐treated cells compared with the PBS‐treated control cells (Figure 6D–G).

### 3.7 DEC2 positively regulates the progression of OS via VEGF signalling in an in vivo mouse model

**FIGURE 7:**
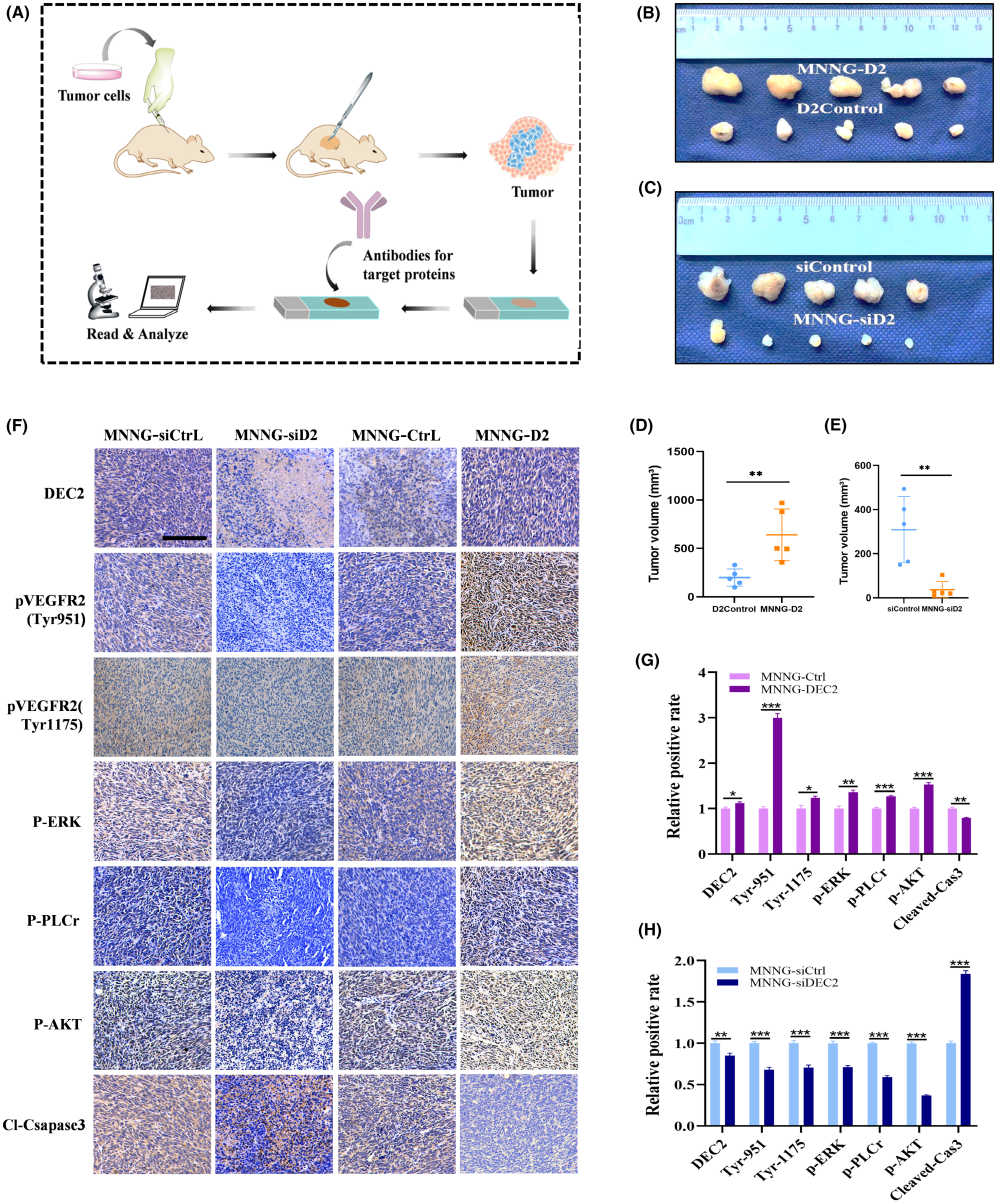
The CDX mouse model confirms DEC2 affects chondroblastic‐like OS cells via VEGF signalling. (A) Diagram illustrating the procedures for the subcutaneous tumour model and IHC analysis. (B, D) Brightfield photomicrographs of excised tumours of MNNG/HOS‐DEC2 (*N* = 5), or DEC2 control cells (*N* = 5) and quantitation of tumour volume (mean ± SD, *N* = 5). (C, E) Brightfield photomicrographs of excised tumours of MNNG/HOS‐siDEC2 (*N* = 5), or siD2control cells (*N* = 5) and quantitation of tumour volume (mean ± SD, *N* = 5). (F) High magnification brightfield images of IHC staining (DAB chromogen) of DEC2, Tyr1175, Tyr951, PAKT, P‐ERK, P‐PLCγ1 and cleaved‐caspase 3 in xenografts of OS tissue from the subcutaneous tumour nude mouse model (scale bars, 200 μm). Counterstain is crystal violet. (G, H) Quantitation of relative positive rates of IHC proteins in tumours (mean ± SD, *N* = 3). *P < 0.05; **P < 0.01; ***P < 0.001; ns, not significant.

Finally, we asked whether the DEC2‐related processes of chondroblastic OS progression we identified in vitro would play out similarly in an in vivo animal model. We used a subcutaneous implantation tumour nude mouse model (Figure 7A). When the longest diameter of the largest tumour reached 200 mm, mice were euthanized, and the tumours were excised, measured, and data recorded. The volume of tumours was calculated as the length (mm) × width (mm)^2^/2. As shown in Figure 7B,D, the growth of the OS implants was significantly enhanced by implanted xenograft of MNNG/HOS‐DEC2 cells (ratio of MNNG/HOS‐DEC2 to DEC2 control was 3.80 ± 1.95), while grafts of MNNG/HOS‐siDEC2 cells resulted in attenuated growth (ratio of MNNG/HOS‐siDEC2 to siDEC2 control was 0.20 ± 0.28) (Figure 7C,E).

We next examined VEGFC/VEGFR2 signalling in xenografts of OS tissue from the mouse model using immunohistochemistry. As shown in Figure 7F, upregulated DEC2 expression in the OS tissue resulted in dense immunostaining of DEC2, VEGFR2_Y1175_, VEGFR2_Y951_, P‐AKT, P‐ERK, P‐PLCγ1 proteins and reduced less dense staining of cleaved‐caspase3 proteins (ratio of MNNG/HOS‐D2 to DEC2 control for DEC2, VEGFR2_Y1175_, VEGFR2_Y951_, P‐AKT, P‐ERK, P‐PLCγ1, and cleaved‐caspase3 was 1.12 ± 0.06, 1.25 ± 0.12, 2.99 ± 0.15, 1.53 ± 0.07, 1.36 ± 0.03, 1.27 ± 0.03, and 0.78 ± 0.02, respectively) (Figure 7G). In the tissue with downregulated DEC2 expression, the staining density of these key proteins showed an opposite pattern (ratio of MNNG/HOS‐siD2 to siD2 Control for DEC2, Tyr1175, Tyr951, P‐AKT, P‐ERK, P‐PLCγ1, and cleaved‐caspase3 was 0.85 ± 0.02, 0.70 ± 0.05, 0.68 ± 0.04, 0.37 ± 0.01, 0.71 ± 0.02, 0.59 ± 0.01, and 1.84 ± 0.07, respectively) (Figure 7H). In summary, these results we obtained in vivo were consistent with the results obtained in vitro.

## 4 DISCUSSION

Despite the heterogeneity of chondroblastic and osteoblastic OS, current therapeutic treatments are similar for both subtypes. Many attempts have been made to develop specific therapies against different types of OS, but little progress has been made for lack of differentiable biomarkers.^9^ Targeted therapies have received considerable attention in clinical trials for their efficacy in the treatment of various kinds of tumours.^34^ However, few studies have been done on the molecular mechanisms of different subtypes of OS. Therefore, there is an urgent need to understand the molecular mechanisms underlying the progression of the different types of OS and to identify specific targets for therapeutic treatment.^35^ Here, we provide multiple lines of evidence that DEC2 distinguishes chondroblastic OS from osteoblastic OS and that DEC2 promotes tumour growth through activation of the VEGFC/VEGFR2 signalling pathway.

DEC2, a transcriptional repressor, has been reported to play a vital role in tumour cell growth under hypoxic conditions.^36^, ^37^ Previous studies also indicate that DEC2 and VEGF expression are related in tumour cell growth. For example, Sato et al^38^ showed that DEC2 negatively regulates VEGF expression by interacting with HIF‐1α in NIH3T3 and Sarcoma 180 cells under hypoxic conditions. Others reported that DEC2 upregulates VEGF gene expression in human Müller cells under hypoxic conditions.^39^ However, few studies have examined the role of site‐specific phosphorylation of VEGFR2 by DEC2, which may trigger different downstream cascades leading to different cell functions.^40^

In the present study, we demonstrated that upregulation of DEC2 was positively correlated with the expression and phosphorylation of VEGFR2 in chondroblastic OS cells. Previous studies confirmed that the Y951 and Y1175 sites of VEGFR2 were the relevant phosphorylation sites.^41^, ^42^ Our results suggest that upregulation of DEC2 specifically activates the VEGFR2_Y1175_ site and its down‐stream cascades, resulting in enhanced cell proliferation of chondroblastic‐like OS cells.

**FIGURE 8:**
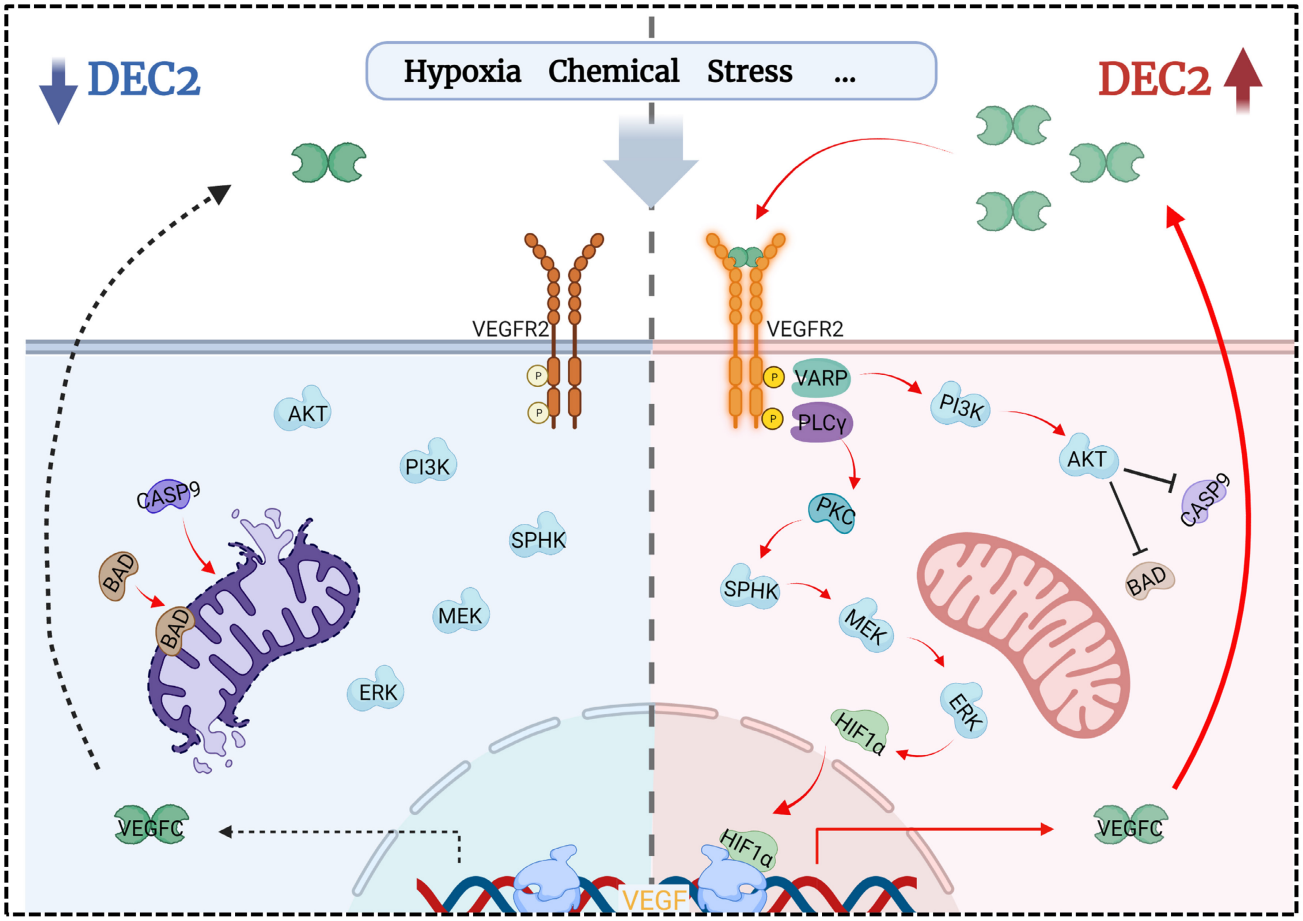
Diagram illustrating the mechanism underlying DEC2 enhancement of cell proliferation and apoptosisresistance via VEGFC/VEGFR2 signalling in chondroblastic osteosarcoma. High expression of DEC2 enhances cell proliferation in chondroblastic‐like OS cells through the VEGFR2Y1175‐PLCγ1‐PKC‐SPHK‐MEK‐ERK signalling pathway. At the same time, DEC2 decreases apoptosis rate via the VEGFR2Y951‐VARP‐PI3K‐AKT signalling pathway and this leads to the inhibition of Caspase9 and Caspase3 activity in chondroblastic‐like OS cells. These two signalling trajectories result in enhanced progression of chondroblastic OS. These differentially expressed genes (DEGs) are shown in Figure 3A,B.

We further examined the activation of the VEGFR2_Y1175_‐PLCγ1 pathway, which previously was reported to mediate cell proliferation in vascular endothelial cells.^43^ Our findings here confirmed that PLCγ1 plays an essential role in cell proliferation through interaction with VEGFR2_Y1175_ in chondroblastic‐like OS cells. Furthermore, VEGFR2_Y1175_‐induced PLCγ1 signalling also activates ERK and HIF‐1, as the downstream cascades of PKC signalling. The activation of HIF‐1 has been previously reported in OS.^15^ Studies have revealed that VEGF is a well‐known downstream target gene of HIF‐1.^44^ We found that upregulation of DEC2 upregulates VEGF‐C expression in chondroblastic OS cells. Taken together, we propose that VEGFC/VEGFR2/HIF‐1/VEGFC plays a positive feedback role in chondroblastic‐like OS cells, which contributes to increased cell proliferation (Figure 8).

Besides the known functions mentioned above, DEC2 also has anti‐apoptotic effects in oesophageal squamous cell carcinoma. breast cancer and prostate cancer.^21^, ^22^, ^45^ This is consistent with results from our present study. We found that upregulation of DEC2 results in a reduced apoptosis rate in chondroblastic‐like OS cells. Moreover, we found that, besides the phosphorylation of Y1175, DEC2 also can phosphorylate the Y951 site of VEGFR2 in chondroblastic‐like OS cells. Wu et al. identified an adapter molecule, designated VEGF receptor‐associated protein (VRAP), as a binding partner to Y951.^46^ Consistent with that observation, we found that VARP can be activated by VEGFR2_Y951_ in chondroblastic‐like OS cells. Furthermore, we also found that PI3K‐Akt was in the down‐stream cascade of VEGFR2_Y951_‐VARP signalling. Specifically, we showed that PI3K‐Akt protein expression was reduced in chondroblastic OS cells after inhibiting VEGFR2_Y951_ with SU5416, which increased cell apoptosis rates. Upregulated DEC2 in our study resulted in decreased cleavage of caspase‐9, caspase‐3 in chondroblastic OS cells, which are signature characteristics of apoptosis. In conclusion, DEC2 inhibited chondroblastic‐like OS cell apoptosis through VEGFR2_Y951_/VARP/Pi3k/AKT/Caspase9/caspase3 pathway (Figure 8).

Although our study has illuminated some of the relationships of DEC2 and the VEGF signalling pathway in chondroblastic OS progression, there are still missing pieces in the puzzle about the underpinnings of chondroblastic OS progression. Firstly, it is still a mystery how VEGFR2 and phosphorylation sites are positively regulated in chondroblastic‐like OS cells under the DEC2 high expressing condition. To address this question, high‐throughput protein–protein interaction screening may need to be performed to discover DEC2 joint proteins that exert their effects in VEGFR2 phosphorylation. Secondly, we only detected the activation of two phosphorylation sites of VEGFR2, which are reported to be highly related to cell growth. Other sites not yet examined require detailed studies in future.

In conclusion, our results demonstrated that high expression of DEC2 distinguishes chondroblastic OS from osteoblastic OS. Moreover, we discovered that high expression of DEC2 in chondroblastic OS cells leads to tumour progression via activation of the VEGFC/VEGFR2 signalling pathway. Thus, targeting the elevated expression of DEC2 could be a therapeutic strategy to treat chondroblastic OS. Decreasing its expression or suppressing the VEGFC/VEGFR2 pathway in chondroblastic OS may be a promising vein to mine for treating patients with this subtype of OS.

## AUTHOR CONTRIBUTIONS

**Maimaitiaili Tuerxun:** Conceptualization (equal); data curation (equal); writing – original draft (lead). **Xu Zheng:** Data curation (equal); formal analysis (equal); investigation (equal); software (equal). **Jun Xu:** Investigation (equal); methodology (equal); resources (equal). **Quanjun Yang:** Formal analysis (equal); methodology (equal); validation (equal). **Ting Yuan:** Data curation (equal); formal analysis (equal); supervision (equal); validation (equal). **Changqing Zhang:** Conceptualization (equal); funding acquisition (equal); methodology (equal); supervision (equal); writing – review and editing (equal). **Shumin Zhou:** Conceptualization (equal); funding acquisition (equal); methodology (equal); project administration (equal); supervision (equal); writing – review and editing (equal).

## FUNDING INFORMATION

This work was supported by grants of the National Natural Science Foundation of China (No. 81820108020), Clinical Research Plan of SHDC (No. SHDC2020CR1025B), Basic Scientific Research Projects of Shanghai Sixth People's Hospital (No. YNMS202202).

## CONFLICT OF INTEREST STATEMENT

The authors declare that the research was conducted in the absence of any commercial or financial relationships that could be construed as a potential conflict of interest.

## Supporting information


Figure S1.

Figure S2.

Figure S3.

Appendix S1.

## Data Availability

The datasets are available from the corresponding author upon reasonable request.
