# Supplementary figures and images for: High expression of DEC2 distinguishes chondroblastic osteosarcoma and promotes tumour growth by activating the VEGFC/VEGFR2 signalling pathway
Source: J Cell Mol Med. 2024 Jun 7;28(11):e18462. doi: 10.1111/jcmm.18462 (PMC11157672; doi:10.1111/jcmm.18462)

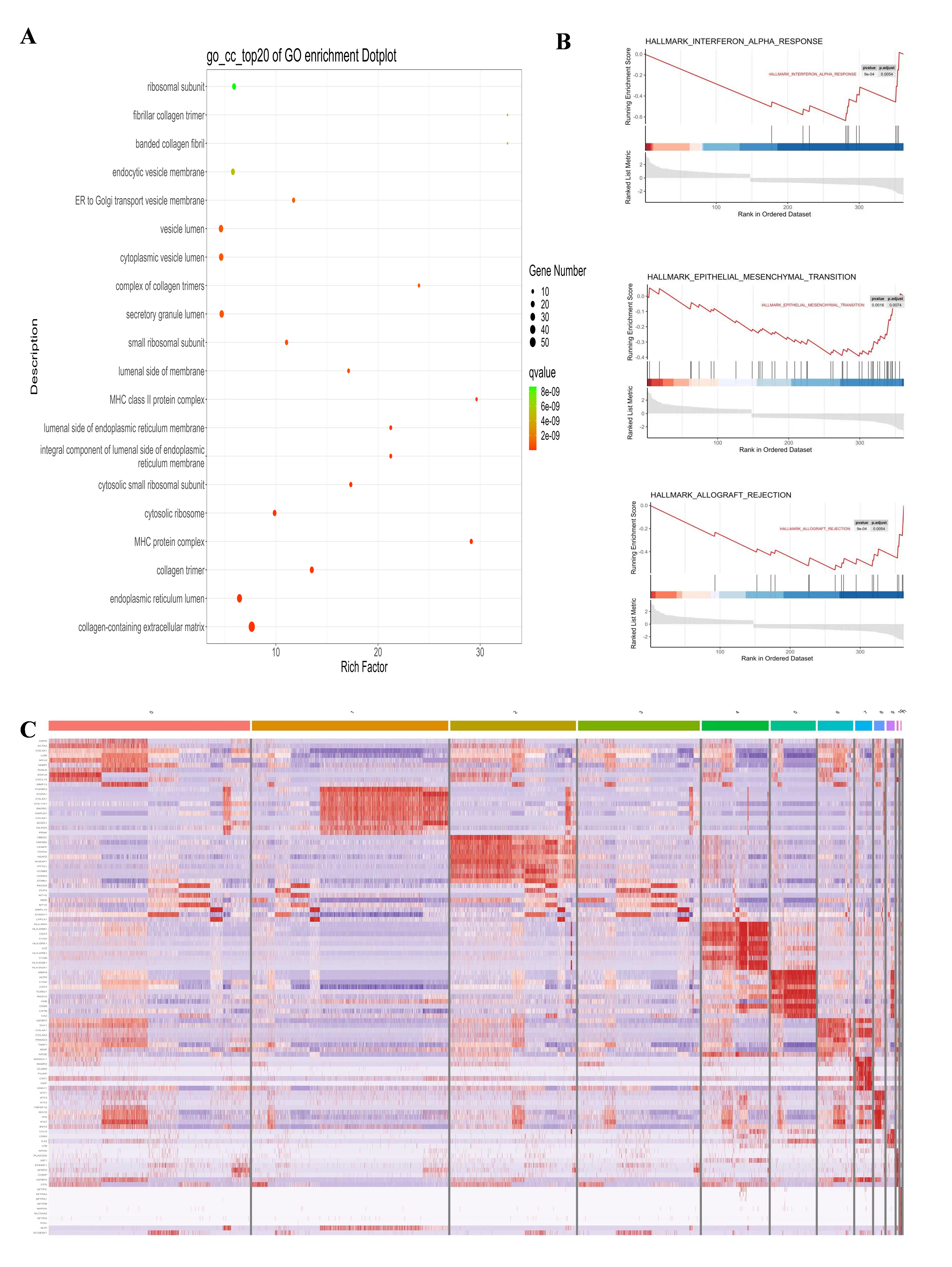

Supplement: Supplementary file 1 — Figure S1. [file JCMM-28-e18462-s003.png]

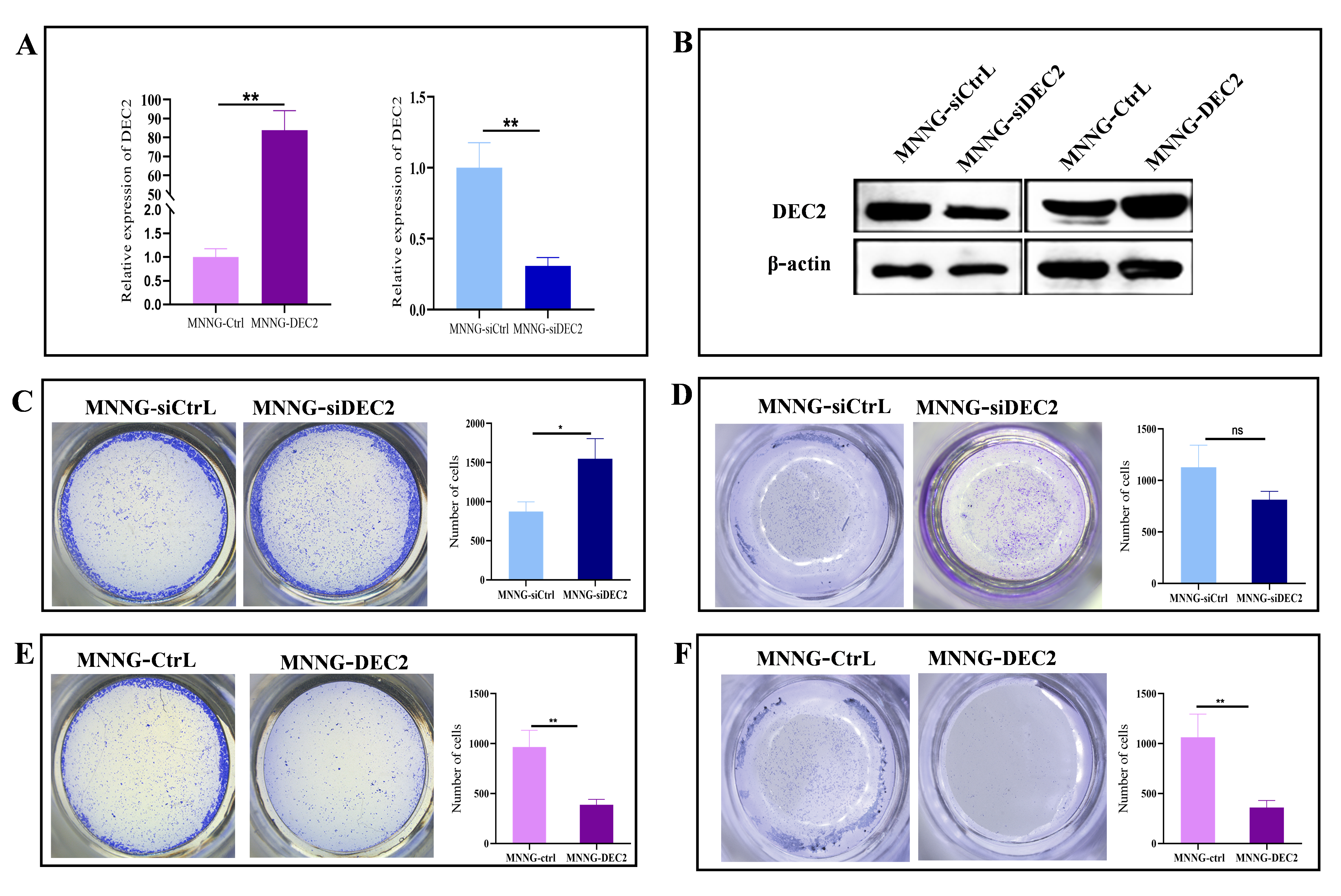

Supplement: Supplementary file 2 — Figure S2. [file JCMM-28-e18462-s004.png]

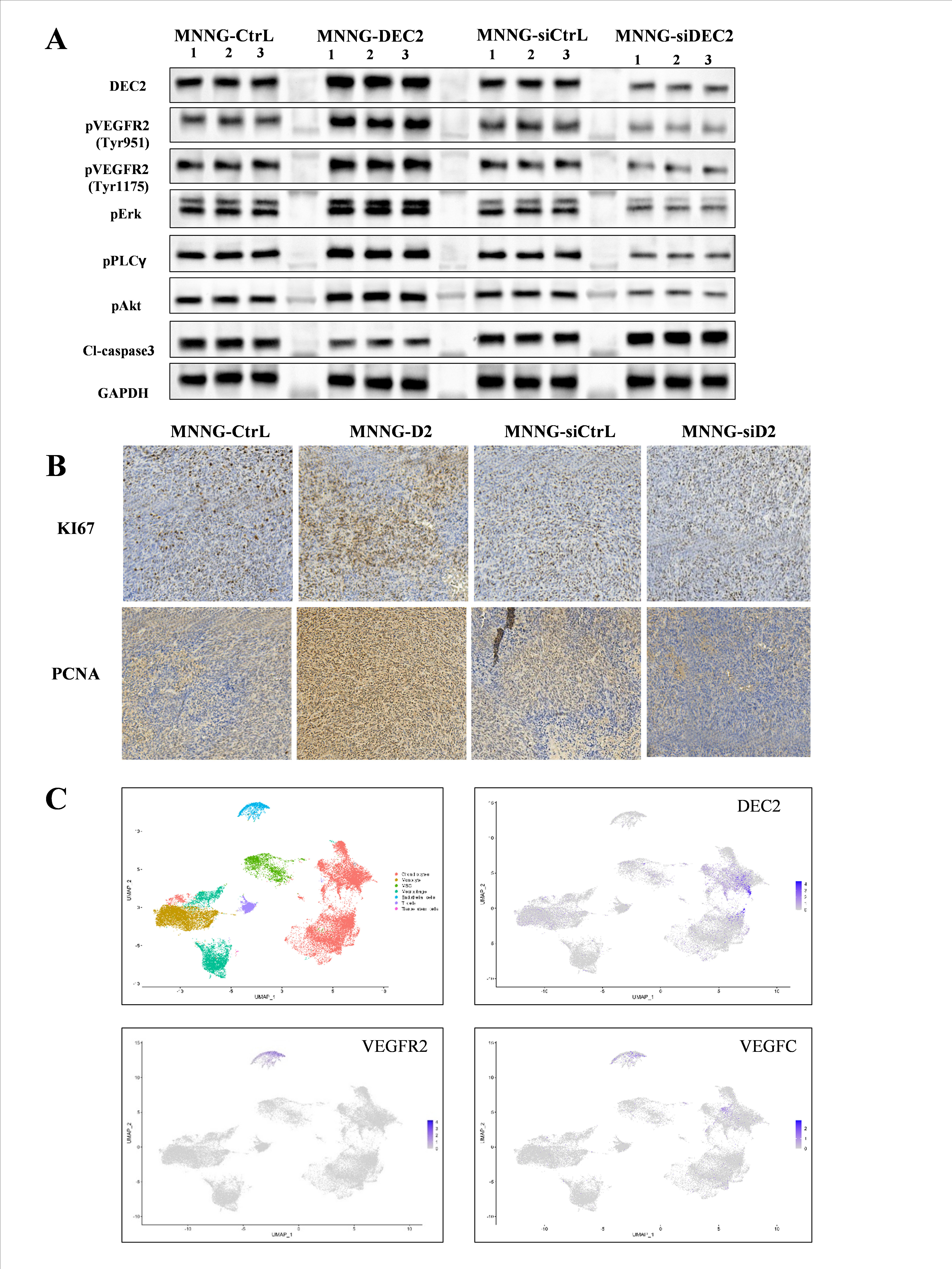

Supplement: Supplementary file 3 — Figure S3. [file JCMM-28-e18462-s002.jpg]
