# Supplementary material for: High expression of DEC2 distinguishes chondroblastic osteosarcoma and promotes tumour growth by activating the VEGFC/VEGFR2 signalling pathway
Source: J Cell Mol Med. 2024 Jun 7;28(11):e18462. doi: 10.1111/jcmm.18462 (PMC11157672; doi:10.1111/jcmm.18462)
Supplement: Supplementary file 4 — Appendix S1. [file JCMM-28-e18462-s001.docx]

**Fig. S1. Clinical characteristics of OS patients and enriched pathways in cell cluster 1. (A)** KEGG pathway enrichment analysis showing the top 20 cellular components in cell cluster 1, as identified in the GO enrichment analysis. **(B)** Three negatively enriched pathways in cell cluster 1. **(C)** Heatmap showing average expression of the top genes in different cell clusters.

**Fig. S2. DEC2 overexpression and DEC2 knockdown verified at transcriptional and translational levels. (A)** Quantitation of DEC2 mRNA in MNNG/HOS-DEC2, MNNG/HOS-siDEC2, and their respective control cells by qRT-PCR (2-ΔΔCt method; N=3). **(B)** Western blot showing DEC2 protein expression levels in MNNG/HOS-DEC2, MNNG/HOS-siDEC2, and their respective control cells. **(C, E)** Results of cell migration assays of MNNG/HOS-DEC2, MNNG/HOS-siDEC2, and their respective control cells. **(D, F)** Results of cell invasion assays of MNNG/HOS-DEC2, MNNG/HOS-siDEC2, and their respective control cells. Data expressed as means±SD (N=3) for all experiments. *P<0.05; **P<0.01; ***P<0.001 for all panels.

**Fig. S3. Verification of protein expression levels in OS tissue from the subcutaneous tumor nude mouse model. (A)** Western blot analysis of protein expression levels of DEC2, Tyr1175, Tyr951, P-AKT, P-ERK, P-PLCγ1, and cleaved-caspase3 in OS tissue from the subcutaneous tumor nude mouse model. (B**)** High magnification brightfield images of IHC staining (DAB chromogen) of KI67, PCNA in xenografts of OS tissue from the subcutaneous tumor nude mouse model. **(C)** Cluster analysis except the OS cells in the same single-cell RNAseq data and the expression of DEC2 , VEGFR2 and VEGFC.

**Supplementary Table 1.** Clinical characteristics of the osteosarcoma patients

| **Sample** | **Pathological type** | **Type** | **Location** | **Necrosis rate** |
| --- | --- | --- | --- | --- |
| BC2 | Conventional | Primary | Femur | <90 |
| BC3 | Conventional | Primary | Tibia | <90 |
| BC5 | Conventional | Primary | Femur | ≥90 |
| BC6 | Conventional | Primary | Ulna | ≥90 |
| BC10 | Conventional | Metastasis | Femur | <90 |
| BC11 | Conventional | Recurrent | Femur | <90 |
| BC16 | Conventional | Primary | Tibia | <90 |
| BC17 | Chondroblastic | Metastasis | Tibia | <90 |
| BC20 | Chondroblastic | Recurrent | Femur | <90 |
| BC21 | Intraosseous osteosarcoma | Primary | Femur | <90 |
| BC22 | Chondroblastic | Primary | Femur | <90 |

**Supplementary Table 2.** Information of the vector and sequences of full-length DEC2 and siRNA against DEC2

|  | **Vector Name** | **Sequences** |
| --- | --- | --- |
| **siRNA against DEC2** | pLKO.5* shControl | CAACAAGATGAAGAGCACCAA |
|  | pLKO.5 shDEC2-1 | CTGGACTATTCCTCTTTGTATAT |
|  | pLKO.5 shDEC2-2 | GTCTTGGAATTAACTTTGAAACA |
|  | pLKO.5 shDEC2-3 | GACCGAATTAATGAATGCATTGC |
| **BHLHE41 (DEC2) (NM_030762.3)** | pLVX-EGFP-IRES-puro# DEC2 | AT GGACGAAGAA ATTCCTCATT TGCAAGAGAG ACAGTTACTG GAACATAGAG ATTTTATAGG ACTGGACTAT TCCTCTTTGT ATATGTGTAA ACCCAAAAGG AGCATGAAAC GAGACGACAC CAAGGATACC TACAAATTAC CGCACAGATT AATAGAAAAG AAAAGAAGAG ACCGAATTAA TGAATGCATT GCTCAGCTGA AAGATTTACT GCCTGAACAT CTGAAATTGA CAACTCTGGG ACATCTGGAG AAAGCTGTAG TCTTGGAATT AACTTTGAAA CACTTAAAAG CTTTAACCGC CTTAACCGAG CAACAGCATC AGAAGATAAT TGCTTTACAG AATGGGGAGC GATCTCTGAA ATCGCCCATT CAGTCCGACT TGGATGCGTT CCACTCGGGA TTTCAAACAT GCGCCAAAGA AGTCTTGCAA TACCTCTCCC GGTTTGAGAG CTGGACACCC AGGGAGCCGC GGTGTGTCCA GCTGATCAAC CACTTGCACG CCGTGGCCAC CCAGTTCTTG CCCACCCCGC AGCTGTTGAC TCAACAGGTC CCTCTGAGCA AAGGCACCGG CGCTCCCTCG GCCGCCGGGT CCGCGGCCGC CCCCTGCCTG GAGCGCGCGG GGCAGAAGCT GGAGCCCCTC GCCTACTGCG TGCCCGTCAT CCAGCGGACT CAGCCCAGCG CCGAGCTCGC CGCCGAGAAC GACACGGACA CCGACAGCGG CTACGGCGGC GAAGCCGAGG CCCGGCCGGA CCGCGAGAAA GGCAAAGGCG CGGGGGCGAG CCGCGTCACC ATCAAGCAGG AGCCTCCCGG GGAGGACTCG CCGGCGCCCA AGCCTCCCGG GGAGGACTCG CCGGCGCCCA AGAGGATGAA GCTGGATTCC CGCGGCGGCG GCAGCGGCGG CGGCCCGGGG GGCGGCGCGG CGGCGGCGGC AGCCGCGCTT CTGGGGCCCG ACCCTGCCGC CGCGGCCGCG CTGCTGAGAC CCGACGCCGC CCTGCTCAGC TCGCTGGTGG CGTTCGGCGG AGGCGGAGGC GCGCCCTTCC CGCAGCCCGC GGCCGCCGCG GCCCCCTTCT GCCTGCCCTT CTGCTTCCTC TCGCCTTCTG CAGCTGCCGC CTACGTGCAG CCCTTCCTGG ACAAGAGCGG CCTGGAGAAG TATCTGTACC CGGCGGCGGC TGCCGCCCCG TTCCCGCTGC TATACCCCGG CATCCCCGCC CCGGCGGCAG CCGCGGCAGC CGCCGCCGCC GCTGCCGCCG CCGCCGCCGC GTTCCCCTGC CTGTCCTCGG TGTTGTCGCC CCCTCCCGAG AAGGCGGGCG CCGCCGCCGC GACCCTCCTG CCGCACGAGG TGGCGCCCCT TGGGGCGCCG CACCCCCAGC ACCCGCACGG CCGCACCCAC CTGCCCTTCG CCGGGCCCCG CGAGCCGGGG AACCCGGAGA GCTCTGCTCA GGAAGATCCC TCGCAGCCAG GAAAGGAAGC TCCCTGA |

***pLKO_TRC005**

CCCGGGTTGCGCCTTTTCCAAGGCAGCCCTGGGTTTGCGCAGGGACGCGGCTGCTCTGGGCGTGGTTCCGGGAAACGCAGCGGCGCCGACCCTGGGTCTCGCACATTCTTCACGTCCGTTCGCAGCGTCACCCGGATCTTCGCCGCTACCCTTGTGGGCCCCCCGGCGACGCTTCCTGCTCCGCCCCTAAGTCGGGAAGGTTCCTTGCGGTTCGCGGCGTGCCGGACGTGACAAACGGAAGCCGCACGTCTCACTAGTACCCTCGCAGACGGACAGCGCCAGGGAGCAATGGCAGCGCGCCGACCGCGATGGGCTGTGGCCAATAGCGGCTGCTCAGCAGGGCGCGCCGAGAGCAGCGGCCGGGAAGGGGCGGTGCGGGAGGCGGGGTGTGGGGCGGTAGTGTGGGCCCTGTTCCTGCCCGCGCGGTGTTCCGCATTCTGCAAGCCTCCGGAGCGCACGTCGGCAGTCGGCTCCCTCGTTGACCGAATCACCGACCTCTCTCCCCAGGGGGATCCACCGGAGCTTACCATGACCGAGTACAAGCCCACGGTGCGCCTCGCCACCCGCGACGACGTCCCCAGGGCCGTACGCACCCTCGCCGCCGCGTTCGCCGACTACCCCGCCACGCGCCACACCGTCGATCCGGACCGCCACATCGAGCGGGTCACCGAGCTGCAAGAACTCTTCCTCACGCGCGTCGGGCTCGACATCGGCAAGGTGTGGGTCGCGGACGACGGCGCCGCCGTGGCGGTCTGGACCACGCCGGAGAGCGTCGAAGCGGGGGCGGTGTTCGCCGAGATCGGCCCGCGCATGGCCGAGTTGAGCGGTTCCCGGCTGGCCGCGCAGCAACAGATGGAAGGCCTCCTGGCGCCGCACCGGCCCAAGGAGCCCGCGTGGTTCCTGGCCACCGTCGGCGTCTCGCCCGACCACCAGGGCAAGGGTCTGGGCAGCGCCGTCGTGCTCCCCGGAGTGGAGGCGGCCGAGCGCGCCGGGGTGCCCGCCTTCCTGGAGACCTCCGCGCCCCGCAACCTCCCCTTCTACGAGCGGCTCGGCTTCACCGTCACCGCCGACGTCGAGGTGCCCGAAGGACCGCGCACCTGGTGCATGACCCGCAAGCCCGGTGCCTGAACGCGTTAAGTCGACAATCAACCTCTGGATTACAAAATTTGTGAAAGATTGACTGGTATTCTTAACTATGTTGCTCCTTTTACGCTATGTGGATACGCTGCTTTAATGCCTTTGTATCATGCTATTGCTTCCCGTATGGCTTTCATTTTCTCCTCCTTGTATAAATCCTGGTTGCTGTCTCTTTATGAGGAGTTGTGGCCCGTTGTCAGGCAACGTGGCGTGGTGTGCACTGTGTTTGCTGACGCAACCCCCACTGGTTGGGGCATTGCCACCACCTGTCAGCTCCTTTCCGGGACTTTCGCTTTCCCCCTCCCTATTGCCACGGCGGAACTCATCGCCGCCTGCCTTGCCCGCTGCTGGACAGGGGCTCGGCTGTTGGGCACTGACAATTCCGTGGTGTTGTCGGGGAAATCATCGTCCTTTCCTTGGCTGCTCGCCTGTGTTGCCACCTGGATTCTGCGCGGGACGTCCTTCTGCTACGTCCCTTCGGCCCTCAATCCAGCGGACCTTCCTTCCCGCGGCCTGCTGCCGGCTCTGCGGCCTCTTCCGCGTCTTCGCCTTCGCCCTCAGACGAGTCGGATCTCCCTTTGGGCCGCCTCCCCGCGTCGACTTTAAGACCAATGACTTACAAGGCAGCTGTAGATCTTAGCCACTTTTTAAAAGAAAAGGGGGGACTGGAAGGGCTAATTCACTCCCAACGAAGACAAGATCTGCTTTTTGCTTGTACTGGGTCTCTCTGGTTAGACCAGATCTGAGCCTGGGAGCTCTCTGGCTAACTAGGGAACCCACTGCTTAAGCCTCAATAAAGCTTGCCTTGAGTGCTTCAAGTAGTGTGTGCCCGTCTGTTGTGTGACTCTGGTAACTAGAGATCCCTCAGACCCTTTTAGTCAGTGTGGAAAATCTCTAGCAGTACGTATAGTAGTTCATGTCATCTTATTATTCAGTATTTATAACTTGCAAAGAAATGAATATCAGAGAGTGAGAGGAACTTGTTTATTGCAGCTTATAATGGTTACAAATAAAGCAATAGCATCACAAATTTCACAAATAAAGCATTTTTTTCACTGCATTCTAGTTGTGGTTTGTCCAAACTCATCAATGTATCTTATCATGTCTGGCTCTAGCTATCCCGCCCCTAACTCCGCCCATCCCGCCCCTAACTCCGCCCAGTTCCGCCCATTCTCCGCCCCATGGCTGACTAATTTTTTTTATTTATGCAGAGGCCGAGGCCGCCTCGGCCTCTGAGCTATTCCAGAAGTAGTGAGGAGGCTTTTTTGGAGGCCTAGGGACGTACCCAATTCGCCCTATAGTGAGTCGTATTACGCGCGCTCACTGGCCGTCGTTTTACAACGTCGTGACTGGGAAAACCCTGGCGTTACCCAACTTAATCGCCTTGCAGCACATCCCCCTTTCGCCAGCTGGCGTAATAGCGAAGAGGCCCGCACCGATCGCCCTTCCCAACAGTTGCGCAGCCTGAATGGCGAATGGGACGCGCCCTGTAGCGGCGCATTAAGCGCGGCGGGTGTGGTGGTTACGCGCAGCGTGACCGCTACACTTGCCAGCGCCCTAGCGCCCGCTCCTTTCGCTTTCTTCCCTTCCTTTCTCGCCACGTTCGCCGGCTTTCCCCGTCAAGCTCTAAATCGGGGGCTCCCTTTAGGGTTCCGATTTAGTGCTTTACGGCACCTCGACCCCAAAAAACTTGATTAGGGTGATGGTTCACGTAGTGGGCCATCGCCCTGATAGACGGTTTTTCGCCCTTTGACGTTGGAGTCCACGTTCTTTAATAGTGGACTCTTGTTCCAAACTGGAACAACACTCAACCCTATCTCGGTCTATTCTTTTGATTTATAAGGGATTTTGCCGATTTCGGCCTATTGGTTAAAAAATGAGCTGATTTAACAAAAATTTAACGCGAATTTTAACAAAATATTAACGCTTACAATTTAGGTGGCACTTTTCGGGGAAATGTGCGCGGAACCCCTATTTGTTTATTTTTCTAAATACATTCAAATATGTATCCGCTCATGAGACAATAACCCTGATAAATGCTTCAATAATATTGAAAAAGGAAGAGTATGAGTATTCAACATTTCCGTGTCGCCCTTATTCCCTTTTTTGCGGCATTTTGCCTTCCTGTTTTTGCTCACCCAGAAACGCTGGTGAAAGTAAAAGATGCTGAAGATCAGTTGGGTGCACGAGTGGGTTACATCGAACTGGATCTCAACAGCGGTAAGATCCTTGAGAGTTTTCGCCCCGAAGAACGTTTTCCAATGATGAGCACTTTTAAAGTTCTGCTATGTGGCGCGGTATTATCCCGTATTGACGCCGGGCAAGAGCAACTCGGTCGCCGCATACACTATTCTCAGAATGACTTGGTTGAGTACTCACCAGTCACAGAAAAGCATCTTACGGATGGCATGACAGTAAGAGAATTATGCAGTGCTGCCATAACCATGAGTGATAACACTGCGGCCAACTTACTTCTGACAACGATCGGAGGACCGAAGGAGCTAACCGCTTTTTTGCACAACATGGGGGATCATGTAACTCGCCTTGATCGTTGGGAACCGGAGCTGAATGAAGCCATACCAAACGACGAGCGTGACACCACGATGCCTGTAGCAATGGCAACAACGTTGCGCAAACTATTAACTGGCGAACTACTTACTCTAGCTTCCCGGCAACAATTAATAGACTGGATGGAGGCGGATAAAGTTGCAGGACCACTTCTGCGCTCGGCCCTTCCGGCTGGCTGGTTTATTGCTGATAAATCTGGAGCCGGTGAGCGTGGGTCTCGCGGTATCATTGCAGCACTGGGGCCAGATGGTAAGCCCTCCCGTATCGTAGTTATCTACACGACGGGGAGTCAGGCAACTATGGATGAACGAAATAGACAGATCGCTGAGATAGGTGCCTCACTGATTAAGCATTGGTAACTGTCAGACCAAGTTTACTCATATATACTTTAGATTGATTTAAAACTTCATTTTTAATTTAAAAGGATCTAGGTGAAGATCCTTTTTGATAATCTCATGACCAAAATCCCTTAACGTGAGTTTTCGTTCCACTGAGCGTCAGACCCCGTAGAAAAGATCAAAGGATCTTCTTGAGATCCTTTTTTTCTGCGCGTAATCTGCTGCTTGCAAACAAAAAAACCACCGCTACCAGCGGTGGTTTGTTTGCCGGATCAAGAGCTACCAACTCTTTTTCCGAAGGTAACTGGCTTCAGCAGAGCGCAGATACCAAATACTGTTCTTCTAGTGTAGCCGTAGTTAGGCCACCACTTCAAGAACTCTGTAGCACCGCCTACATACCTCGCTCTGCTAATCCTGTTACCAGTGGCTGCTGCCAGTGGCGATAAGTCGTGTCTTACCGGGTTGGACTCAAGACGATAGTTACCGGATAAGGCGCAGCGGTCGGGCTGAACGGGGGGTTCGTGCACACAGCCCAGCTTGGAGCGAACGACCTACACCGAACTGAGATACCTACAGCGTGAGCTATGAGAAAGCGCCACGCTTCCCGAAGGGAGAAAGGCGGACAGGTATCCGGTAAGCGGCAGGGTCGGAACAGGAGAGCGCACGAGGGAGCTTCCAGGGGGAAACGCCTGGTATCTTTATAGTCCTGTCGGGTTTCGCCACCTCTGACTTGAGCGTCGATTTTTGTGATGCTCGTCAGGGGGGCGGAGCCTATGGAAAAACGCCAGCAACGCGGCCTTTTTACGGTTCCTGGCCTTTTGCTGGCCTTTTGCTCACATGTTCTTTCCTGCGTTATCCCCTGATTCTGTGGATAACCGTATTACCGCCTTTGAGTGAGCTGATACCGCTCGCCGCAGCCGAACGACCGAGCGCAGCGAGTCAGTGAGCGAGGAAGCGGAAGAGCGCCCAATACGCAAACCGCCTCTCCCCGCGCGTTGGCCGATTCATTAATGCAGCTGGCACGACAGGTTTCCCGACTGGAAAGCGGGCAGTGAGCGCAACGCAATTAATGTGAGTTAGCTCACTCATTAGGCACCCCAGGCTTTACACTTTATGCTTCCGGCTCGTATGTTGTGTGGAATTGTGAGCGGATAACAATTTCACACAGGAAACAGCTATGACCATGATTACGCCAAGCGCGCAATTAACCCTCACTAAAGGGAACAAAAGCTGGAGCTGCAAGCTTAATGTAGTCTTATGCAATACTCTTGTAGTCTTGCAACATGGTAACGATGAGTTAGCAACATGCCTTACAAGGAGAGAAAAAGCACCGTGCATGCCGATTGGTGGAAGTAAGGTGGTACGATCGTGCCTTATTAGGAAGGCAACAGACGGGTCTGACATGGATTGGACGAACCACTGAATTGCCGCATTGCAGAGATATTGTATTTAAGTGCCTAGCTCGATACATAAACGGGTCTCTCTGGTTAGACCAGATCTGAGCCTGGGAGCTCTCTGGCTAACTAGGGAACCCACTGCTTAAGCCTCAATAAAGCTTGCCTTGAGTGCTTCAAGTAGTGTGTGCCCGTCTGTTGTGTGACTCTGGTAACTAGAGATCCCTCAGACCCTTTTAGTCAGTGTGGAAAATCTCTAGCAGTGGCGCCCGAACAGGGACTTGAAAGCGAAAGGGAAACCAGAGGAGCTCTCTCGACGCAGGACTCGGCTTGCTGAAGCGCGCACGGCAAGAGGCGAGGGGCGGCGACTGGTGAGTACGCCAAAAATTTTGACTAGCGGAGGCTAGAAGGAGAGAGATGGGTGCGAGAGCGTCAGTATTAAGCGGGGGAGAATTAGATCGCGATGGGAAAAAATTCGGTTAAGGCCAGGGGGAAAGAAAAAATATAAATTAAAACATATAGTATGGGCAAGCAGGGAGCTAGAACGATTCGCAGTTAATCCTGGCCTGTTAGAAACATCAGAAGGCTGTAGACAAATACTGGGACAGCTACAACCATCCCTTCAGACAGGATCAGAAGAACTTAGATCATTATATAATACAGTAGCAACCCTCTATTGTGTGCATCAAAGGATAGAGATAAAAGACACCAAGGAAGCTTTAGACAAGATAGAGGAAGAGCAAAACAAAAGTAAGACCACCGCACAGCAAGCGGCCGCTGATCTTCAGACCTGGAGGAGGAGATATGAGGGACAATTGGAGAAGTGAATTATATAAATATAAAGTAGTAAAAATTGAACCATTAGGAGTAGCACCCACCAAGGCAAAGAGAAGAGTGGTGCAGAGAGAAAAAAGAGCAGTGGGAATAGGAGCTTTGTTCCTTGGGTTCTTGGGAGCAGCAGGAAGCACTATGGGCGCAGCGTCAATGACGCTGACGGTACAGGCCAGACAATTATTGTCTGGTATAGTGCAGCAGCAGAACAATTTGCTGAGGGCTATTGAGGCGCAACAGCATCTGTTGCAACTCACAGTCTGGGGCATCAAGCAGCTCCAGGCAAGAATCCTGGCTGTGGAAAGATACCTAAAGGATCAACAGCTCCTGGGGATTTGGGGTTGCTCTGGAAAACTCATTTGCACCACTGCTGTGCCTTGGAATGCTAGTTGGAGTAATAAATCTCTGGAACAGATTTGGAATCACACGACCTGGATGGAGTGGGACAGAGAAATTAACAATTACACAAGCTTAATACACTCCTTAATTGAAGAATCGCAAAACCAGCAAGAAAAGAATGAACAAGAATTATTGGAATTAGATAAATGGGCAAGTTTGTGGAATTGGTTTAACATAACAAATTGGCTGTGGTATATAAAATTATTCATAATGATAGTAGGAGGCTTGGTAGGTTTAAGAATAGTTTTTGCTGTACTTTCTATAGTGAATAGAGTTAGGCAGGGATATTCACCATTATCGTTTCAGACCCACCTCCCAACCCCGAGGGGACCCAGAGAGGGCCTATTTCCCATGATTCCTTCATATTTGCATATACGATACAAGGCTGTTAGAGAGATAATTAGAATTAATTTGACTGTAAACACAAAGATATTAGTACAAAATACGTGACGTAGAAAGTAATAATTTCTTGGGTAGTTTGCAGTTTTAAAATTATGTTTTAAAATGGACTATCATATGCTTACCGTAACTTGAAAGTATTTCGATTTCTTGGCTTTATATATCTTGTGGAAAGGACGAGGTACCGGTGTGTTGTAAATGAGCACACAAAATACACATGCTAAAATATTATATTCTATGACCTTTATAAAATCAACCAAAATCTTCTTTTTAATAACTTTAGTATCAATAATTAGAATTTTTATGTTCCTTTTTGCAAACTTTTAATAAAAATGAGCAAAATAAAAAAACGCTAGTTTTAGTAACTCGCGTTGTTTTCTTCACCTTTAATAATAGCTACTCCACCACTTGTTCCTAAGCGGTCAGCTCCTGCTTCAATCATTTTTTGAGCATCTTCAAATGTTCTAACTCCACCAGCTGCTTTAACTAAAGCATTGTCTTTAACAACTGACTTCATTAGTTTAACATCTTCAAATGTTGCACCTGATTTTGAAAATCCTGTTGATGTTTTAACAAATTCTAATCCAGCTTCAACAGCTATTTCACAAGCTTTCATGATTTCTTCTTTTGTTAATAAACAATTTTCCATAATACATTTAACAACATGTGATCCAGCTGCTTTTTTTACAGCTTTCATGTCTTCTAAAACTAATTCATAATTTTTGTCTTTTAATGCACCAATATTTAATACCATATCAATTTCTGTTGCACCATCTTTAATTGCTTCAGAAACTTCGAATGCTTTTGTAGCTGTTGTGCATGCACCTAGAGGAAAACCTACAACATTTGTTATTCCTACATTTGTGCCTTTTAATAATTCTTTACAATAGCTTGTTCAATATGAATTAACACAAACTGTTGCAAAATCAAATTCAATTGCTTCATCACATAATTGTTTAATTTCAGCTTTCGTAGCATCTTGTTTTAATAATGTGTGATCTATATATTTGTTTAGTTTCATTTTTTCTCCTATATATTCATTTTTAATTTTAATTCTTTAATAATTTCGTCTACTTTAACTTTAGCGTTTTGAACAGATTCACCAACACCTATAAAATAAATTTTTAGTTTAGGTTCAGTTCCACTTGGGCGAACAGCAAATCATGACTTATCTTCTAAATAAAATTTTAGTAAGTCTTGTCCTGGCATATTATACATTCCATCGATGTAGTCTTCAACATTAACAACTTTAAGTCCAGCAATTTGAGTTAAGGGTGTTGCTCTCAATGATTTCATTAATGGTTCAATTTTTAATTTCTTTTCTTCTGGTTTAAAATTCAAGTTTAAAGTGAAAGTGTAATATGCACCCATTTCTTTAAATAAATCTTCTAAATAGTCTACTAATGTTTTATTTTGTTTTTTATAAAATCAAGCAGCCTCTGCTATTAATATAGAAGCTTGTATTCCATCTTTATCTCTAGCTGAGTCATCAAT

TACATATCCATAACTTTCTTCATAAGCAAAAACAAAATTTAATCCGTTATCTTCTTCTTTAGCAATTTCTCTACCCATTCATTTAAATCCAGTTAAAGTTTTTACAATATTAACTCCATATTTTTCATGAGCGATTCTATCACCCAAATCACTTGTTACAAAACTTGAATATAGAGCCGGATTTTTTGGAATGCTATTTAAGCGTTTTAGATTTGATAATTTTCAATCAATTAAAATTGGTCCTGTTTGATTTCCATCTAATCTTACAAAATGACCATCATGTTTTATTGCCATTCCAAATCTGTCAGCATCTGGGTCATTCATAATAATAATATCTGCATCATGTTTAATACCATATTCAAGCGGTATTTTTCATGCAGGATCAAATTCTGGATTTGGATTTACAACATTTTTAAATGTTTCATCTTCAAATGCATGCTCTTCAACCTCAATAACGTTATATCCTGATTCACGTAATATTTTTGGGGTAAATTTAGTTCCTGTTCCATTAACTGCGCTAAAAATAATTTTTAAATCTTTTTTAGCTTCTTGCTCTTTTTTGTAGAATTCTAGATCTTGAGACAAATGGCAGTATTCATCCACAATTTTAAAAGAAAAGGGGGGATTGGGGGGTACAGTGCAGGGGAAAGAATAGTAGACATAATAGCAACAGACATACAAACTAAAGAATTACAAAAACAAATTACAAAAATTCAAAATTTTCGGGTTTATTACAGGGACAGCAGAGATCCACTTTGGCGCCGGCTCGAGGGGG

**#pLVX-EGFP-IRES-puro Sequences**

TGGAAGGGCTAATTCACTCCCAAAGAAGACAAGATATCCTTGATCTGTGGATCTACCACACACAAGGCTACTTCCCTGATTAGCAGAACTACACACCAGGGCCAGGGGTCAGATATCCACTGACCTTTGGATGGTGCTACAAGCTAGTACCAGTTGAGCCAGATAAGGTAGAAGAGGCCAATAAAGGAGAGAACACCAGCTTGTTACACCCTGTGAGCCTGCATGGGATGGATGACCCGGAGAGAGAAGTGTTAGAGTGGAGGTTTGACAGCCGCCTAGCATTTCATCACGTGGCCCGAGAGCTGCATCCGGAGTACTTCAAGAACTGCTGATATCGAGCTTGCTACAAGGGACTTTCCGCTGGGGACTTTCCAGGGAGGCGTGGCCTGGGCGGGACTGGGGAGTGGCGAGCCCTCAGATCCTGCATATAAGCAGCTGCTTTTTGCCTGTACTGGGTCTCTCTGGTTAGACCAGATCTGAGCCTGGGAGCTCTCTGGCTAACTAGGGAACCCACTGCTTAAGCCTCAATAAAGCTTGCCTTGAGTGCTTCAAGTAGTGTGTGCCCGTCTGTTGTGTGACTCTGGTAACTAGAGATCCCTCAGACCCTTTTAGTCAGTGTGGAAAATCTCTAGCAGTGGCGCCCGAACAGGGACTTGAAAGCGAAAGGGAAACCAGAGGAGCTCTCTCGACGCAGGACTCGGCTTGCTGAAGCGCGCACGGCAAGAGGCGAGGGGCGGCGACTGGTGAGTACGCCAAAAATTTTGACTAGCGGAGGCTAGAAGGAGAGAGATGGGTGCGAGAGCGTCAGTATTAAGCGGGGGAGAATTAGATCGCGATGGGAAAAAATTCGGTTAAGGCCAGGGGGAAAGAAAAAATATAAATTAAAACATATAGTATGGGCAAGCAGGGAGCTAGAACGATTCGCAGTTAATCCTGGCCTGTTAGAAACATCAGAAGGCTGTAGACAAATACTGGGACAGCTACAACCATCCCTTCAGACAGGATCAGAAGAACTTAGATCATTATATAATACAGTAGCAACCCTCTATTGTGTGCATCAAAGGATAGAGATAAAAGACACCAAGGAAGCTTTAGACAAGATAGAGGAAGAGCAAAACAAAAGTAAGACCACCGCACAGCAAGCGGCCGGCCGCTGATCTTCAGACCTGGAGGAGGAGATATGAGGGACAATTGGAGAAGTGAATTATATAAATATAAAGTAGTAAAAATTGAACCATTAGGAGTAGCACCCACCAAGGCAAAGAGAAGAGTGGTGCAGAGAGAAAAAAGAGCAGTGGGAATAGGAGCTTTGTTCCTTGGGTTCTTGGGAGCAGCAGGAAGCACTATGGGCGCAGCGTCAATGACGCTGACGGTACAGGCCAGACAATTATTGTCTGGTATAGTGCAGCAGCAGAACAATTTGCTGAGGGCTATTGAGGCGCAACAGCATCTGTTGCAACTCACAGTCTGGGGCATCAAGCAGCTCCAGGCAAGAATCCTGGCTGTGGAAAGATACCTAAAGGATCAACAGCTCCTGGGGATTTGGGGTTGCTCTGGAAAACTCATTTGCACCACTGCTGTGCCTTGGAATGCTAGTTGGAGTAATAAATCTCTGGAACAGATTTGGAATCACACGACCTGGATGGAGTGGGACAGAGAAATTAACAATTACACAAGCTTAATACACTCCTTAATTGAAGAATCGCAAAACCAGCAAGAAAAGAATGAACAAGAATTATTGGAATTAGATAAATGGGCAAGTTTGTGGAATTGGTTTAACATAACAAATTGGCTGTGGTATATAAAATTATTCATAATGATAGTAGGAGGCTTGGTAGGTTTAAGAATAGTTTTTGCTGTACTTTCTATAGTGAATAGAGTTAGGCAGGGATATTCACCATTATCGTTTCAGACCCACCTCCCAACCCCGAGGGGACCCGACAGGCCCGAAGGAATAGAAGAAGAAGGTGGAGAGAGAGACAGAGACAGATCCATTCGATTAGTGAACGGATCTCGACGGTATCGCCTTTAAAAGAAAAGGGGGGATTGGGGGGTACAGTGCAGGGGAAAGAATAGTAGACATAATAGCAACAGACATACAAACTAAAGAATTACAAAAACAAATTACAAAAATTCAAAATTTTCGGGTTTATTACAGGGACAGCAGAGATCCAGTTTATCGATAAGCTTGGGAGTTCCGCGTTACATAACTTACGGTAAATGGCCCGCCTGGCTGACCGCCCAACGACCCCCGCCCATTGACGTCAATAATGACGTATGTTCCCATAGTAACGCCAATAGGGACTTTCCATTGACGTCAATGGGTGGAGTATTTACGGTAAACTGCCCACTTGGCAGTACATCAAGTGTATCATATGCCAAGTACGCCCCCTATTGACGTCAATGACGGTAAATGGCCCGCCTGGCATTATGCCCAGTACATGACCTTATGGGACTTTCCTACTTGGCAGTACATCTACGTATTAGTCATCGCTATTACCATGGTGATGCGGTTTTGGCAGTACATCAATGGGCGTGGATAGCGGTTTGACTCACGGGGATTTCCAAGTCTCCACCCCATTGACGTCAATGGGAGTTTGTTTTGGCACCAAAATCAACGGGACTTTCCAAAATGTCGTAACAACTCCGCCCCATTGACGCAAATGGGCGGTAGGCGTGTACGGTGGGAGGTCTATATAAGCAGAGCTCGTTTAGTGAACCGTCAGATCGCCTGGAGACGCCATCCACGCTGTTTTGACCTCCATAGAAGACACCGACTCTACTAGAGGATCTATTTCCGGTGAATTCCTCGAGACTAGATCAACAAGTTTGTACAAAAAAGCAGGCTCCGCGGCCGCCCCCTTCACCATGGTGAGCAAGGGCGAGGAGCTGTTCACCGGGGTGGTGCCCATCCTGGTCGAGCTGGACGGCGACGTAAACGGCCACAAGTTCAGCGTGTCCGGCGAGGGCGAGGGCGATGCCACCTACGGCAAGCTGACCCTGAAGTTCATCTGCACCACCGGCAAGCTGCCCGTGCCCTGGCCCACCCTCGTGACCACCCTGACCTACGGCGTGCAGTGCTTCAGCCGCTACCCCGACCACATGAAGCAGCACGACTTCTTCAAGTCCGCCATGCCCGAAGGCTACGTCCAGGAGCGCACCATCTTCTTCAAGGACGACGGCAACTACAAGACCCGCGCCGAGGTGAAGTTCGAGGGCGACACCCTGGTGAACCGCATCGAGCTGAAGGGCATCGACTTCAAGGAGGACGGCAACATCCTGGGGCACAAGCTGGAGTACAACTACAACAGCCACAACGTCTATATCATGGCCGACAAGCAGAAGAACGGCATCAAGGTGAACTTCAAGATCCGCCACAACATCGAGGACGGCAGCGTGCAGCTCGCCGACCACTACCAGCAGAACACCCCCATCGGCGACGGCCCCGTGCTGCTGCCCGACAACCACTACCTGAGCACCCAGTCCGCCCTGAGCAAAGACCCCAACGAGAAGCGCGATCACATGGTCCTGCTGGAGTTCGTGACCGCCGCCGGGATCACTCTCGGCATGGACGAGCTGTACAAGTAAAAGGGTGGGCGCGCCGACCCAGCTTTCTTGTACAAAGTGGTTGATCTAGTTCTAGAGCGGCCGCGGATCCCGCCCCTCTCCCTCCCCCCCCCCTAACGTTACTGGCCGAAGCCGCTTGGAATAAGGCCGGTGTGCGTTTGTCTATATGTTATTTTCCACCATATTGCCGTCTTTTGGCAATGTGAGGGCCCGGAAACCTGGCCCTGTCTTCTTGACGAGCATTCCTAGGGGTCTTTCCCCTCTCGCCAAAGGAATGCAAGGTCTGTTGAATGTCGTGAAGGAAGCAGTTCCTCTGGAAGCTTCTTGAAGACAAACAACGTCTGTAGCGACCCTTTGCAGGCAGCGGAACCCCCCACCTGGCGACAGGTGCCTCTGCGGCCAAAAGCCACGTGTATAAGATACACCTGCAAAGGCGGCACAACCCCAGTGCCACGTTGTGAGTTGGATAGTTGTGGAAAGAGTCAAATGGCTCTCCTCAAGCGTATTCAACAAGGGGCTGAAGGATGCCCAGAAGGTACCCCATTGTATGGGATCTGATCTGGGGCCTCGGTGCACATGCTTTACATGTGTTTAGTCGAGGTTAAAAAAACGTCTAGGCCCCCCGAACCACGGGGACGTGGTTTTCCTTTGAAAAACACGATGATAAGCTTGCCACAACCCACAAGGAGACGACCTTCCATGACCGAGTACAAGCCCACGGTGCGCCTCGCCACCCGCGACGACGTCCCCCGGGCCGTACGCACCCTCGCCGCCGCGTTCGCCGACTACCCCGCCACGCGCCACACCGTCGACCCGGACCGCCACATCGAGCGGGTCACCGAGCTGCAAGAACTCTTCCTCACGCGCGTCGGGCTCGACATCGGCAAGGTGTGGGTCGCGGACGACGGCGCCGCGGTGGCGGTCTGGACCACGCCGGAGAGCGTCGAAGCGGGGGCGGTGTTCGCCGAGATCGGCCCGCGCATGGCCGAGTTGAGCGGTTCCCGGCTGGCCGCGCAGCAACAGATGGAAGGCCTCCTGGCGCCGCACCGGCCCAAGGAGCCCGCGTGGTTCCTGGCCACCGTCGGCGTCTCGCCCGACCACCAGGGCAAGGGTCTGGGCAGCGCCGTCGTGCTCCCCGGAGTGGAGGCGGCCGAGCGCGCCGGGGTGCCCGCCTTCCTGGAGACCTCCGCGCCCCGCAACCTCCCCTTCTACGAGCGGCTCGGCTTCACCGTCACCGCCGACGTCGAGGTGCCCGAAGGACCGCGCACCTGGTGCATGACCCGCAAGCCCGGTGCCTAGACGCGTCTGGAACAATCAACCTCTGGATTACAAAATTTGTGAAAGATTGACTGGTATTCTTAACTATGTTGCTCCTTTTACGCTATGTGGATACGCTGCTTTAATGCCTTTGTATCATGCTATTGCTTCCCGTATGGCTTTCATTTTCTCCTCCTTGTATAAATCCTGGTTGCTGTCTCTTTATGAGGAGTTGTGGCCCGTTGTCAGGCAACGTGGCGTGGTGTGCACTGTGTTTGCTGACGCAACCCCCACTGGTTGGGGCATTGCCACCACCTGTCAGCTCCTTTCCGGGACTTTCGCTTTCCCCCTCCCTATTGCCACGGCGGAACTCATCGCCGCCTGCCTTGCCCGCTGCTGGACAGGGGCTCGGCTGTTGGGCACTGACAATTCCGTGGTGTTGTCGGGGAAGCTGACGTCCTTTCCATGGCTGCTCGCCTGTGTTGCCACCTGGATTCTGCGCGGGACGTCCTTCTGCTACGTCCCTTCGGCCCTCAATCCAGCGGACCTTCCTTCCCGCGGCCTGCTGCCGGCTCTGCGGCCTCTTCCGCGTCTTCGCCTTCGCCCTCAGACGAGTCGGATCTCCCTTTGGGCCGCCTCCCCGCCTGGAATTAATTCTGCAGTCGAGACCTAGAAAAACATGGAGCAATCACAAGTAGCAATACAGCAGCTACCAATGCTGATTGTGCCTGGCTAGAAGCACAAGAGGAGGAGGAGGTGAGTTTTCCAGTCACACCTCAGGTACCTTTAAGACCAATGACTTACAAGGCAGCTGTAGATCTTAGCCACTTTTTAAAAGAAAAGAGGGGACTGGAAGGGCTAATTCACTCCCAACGAAGACAAGATATCCTTGATCTGTGGATCTACCACACACAAGGCTACTTCCCTGATTAGCAGAACTACACACCAGGGCCAGGGGTCAGATATCCACTGACCTTTGGATGGTGCTACAAGCTAGTACCAGTTGAGCCAGATAAGGTAGAAGAGGCCAATAAAGGAGAGAACACCAGCTTGTTACACCCTGTGAGCCTGCATGGGATGGATGACCCGGAGAGAGAAGTGTTAGAGTGGAGGTTTGACAGCCGCCTAGCATTTCATCACGTGGCCCGAGAGCTGCATCCGGAGTACTTCAAGAACTGCTGATATCGAGCTTGCTACAAGGGACTTTCCGCTGGGGACTTTCCAGGGAGGCGTGGCCTGGGCGGGACTGGGGAGTGGCGAGCCCTCAGATCCTGCATATAAGCAGCTGCTTTTTGCCTGTACTGGGTCTCTCTGGTTAGACCAGATCTGAGCCTGGGAGCTCTCTGGCTAACTAGGGAACCCACTGCTTAAGCCTCAATAAAGCTTGCCTTGAGTGCTTCAAGTAGTGTGTGCCCGTCTGTTGTGTGACTCTGGTAACTAGAGATCCCTCAGACCCTTTTAGTCAGTGTGGAAAATCTCTAGCAGTAGTAGTTCATGTCATCTTATTATTCAGTATTTATAACTTGCAAAGAAATGAATATCAGAGAGTGAGAGGCCTTGACATTGCTAGCGTTTACCGTCGACCTCTAGCTAGAGCTTGGCGTAATCATGGTCATAGCTGTTTCCTGTGTGAAATTGTTATCCGCTCACAATTCCACACAACATACGAGCCGGAAGCATAAAGTGTAAAGCCTGGGGTGCCTAATGAGTGAGCTAACTCACATTAATTGCGTTGCGCTCACTGCCCGCTTTCCAGTCGGGAAACCTGTCGTGCCAGCTGCATTAATGAATCGGCCAACGCGCGGGGAGAGGCGGTTTGCGTATTGGGCGCTCTTCCGCTTCCTCGCTCACTGACTCGCTGCGCTCGGTCGTTCGGCTGCGGCGAGCGGTATCAGCTCACTCAAAGGCGGTAATACGGTTATCCACAGAATCAGGGGATAACGCAGGAAAGAACATGTGAGCAAAAGGCCAGCAAAAGGCCAGGAACCGTAAAAAGGCCGCGTTGCTGGCGTTTTTCCATAGGCTCCGCCCCCCTGACGAGCATCACAAAAATCGACGCTCAAGTCAGAGGTGGCGAAACCCGACAGGACTATAAAGATACCAGGCGTTTCCCCCTGGAAGCTCCCTCGTGCGCTCTCCTGTTCCGACCCTGCCGCTTACCGGATACCTGTCCGCCTTTCTCCCTTCGGGAAGCGTGGCGCTTTCTCATAGCTCACGCTGTAGGTATCTCAGTTCGGTGTAGGTCGTTCGCTCCAAGCTGGGCTGTGTGCACGAACCCCCCGTTCAGCCCGACCGCTGCGCCTTATCCGGTAACTATCGTCTTGAGTCCAACCCGGTAAGACACGACTTATCGCCACTGGCAGCAGCCACTGGTAACAGGATTAGCAGAGCGAGGTATGTAGGCGGTGCTACAGAGTTCTTGAAGTGGTGGCCTAACTACGGCTACACTAGAAGAACAGTATTTGGTATCTGCGCTCTGCTGAAGCCAGTTACCTTCGGAAAAAGAGTTGGTAGCTCTTGATCCGGCAAACAAACCACCGCTGGTAGCGGTGGTTTTTTTGTTTGCAAGCAGCAGATTACGCGCAGAAAAAAAGGATCTCAAGAAGATCCTTTGATCTTTTCTACGGGGTCTGACGCTCAGTGGAACGAAAACTCACGTTAAGGGATTTTGGTCATGAGATTATCAAAAAGGATCTTCACCTAGATCCTTTTAAATTAAAAATGAAGTTTTAAATCAATCTAAAGTATATATGAGTAAACTTGGTCTGACAGTTACCAATGCTTAATCAGTGAGGCACCTATCTCAGCGATCTGTCTATTTCGTTCATCCATAGTTGCCTGACTCCCCGTCGTGTAGATAACTACGATACGGGAGGGCTTACCATCTGGCCCCAGTGCTGCAATGATACCGCGAGACCCACGCTCACCGGCTCCAGATTTATCAGCAATAAACCAGCCAGCCGGAAGGGCCGAGCGCAGAAGTGGTCCTGCAACTTTATCCGCCTCCATCCAGTCTATTAATTGTTGCCGGGAAGCTAGAGTAAGTAGTTCGCCAGTTAATAGTTTGCGCAACGTTGTTGCCATTGCTACAGGCATCGTGGTGTCACGCTCGTCGTTTGGTATGGCTTCATTCAGCTCCGGTTCCCAACGATCAAGGCGAGTTACATGATCCCCCATGTTGTGCAAAAAAGCGGTTAGCTCCTTCGGTCCTCCGATCGTTGTCAGAAGTAAGTTGGCCGCAGTGTTATCACTCATGGTTATGGCAGCACTGCATAATTCTCTTACTGTCATGCCATCCGTAAGATGCTTTTCTGTGACTGGTGAGTACTCAACCAAGTCATTCTGAGAATAGTGTATGCGGCGACCGAGTTGCTCTTGCCCGGCGTCAATACGGGATAATACCGCGCCACATAGCAGAACTTTAAAAGTGCTCATCATTGGAAAACGTTCTTCGGGGCGAAAACTCTCAAGGATCTTACCGCTGTTGAGATCCAGTTCGATGTAACCCACTCGTGCACCCAACTGATCTTCAGCATCTTTTACTTTCACCAGCGTTTCTGGGTGAGCAAAAACAGGAAGGCAAAATGCCGCAAAAAAGGGAATAAGGGCGACACGGAAATGTTGAATACTCATACTCTTCCTTTTTCAATATTATTGAAGCATTTATCAGGGTTATTGTCTCATGAGCGGATACATATTTGAATGTATTTAGAAAAATAAACAAATAGGGGTTCCGCGCACATTTCCCCGAAAAGTGCCACCTGACGTCGACGGATCGGGAGATCAACTTGTTTATTGCAGCTTATAATGGTTACAAATAAAGCAATAGCATCACAAATTTCACAAATAAAGCATTTTTTTCACTGCATTCTAGTTGTGGTTTGTCCAAACTCATCAATGTATCTTATCATGTCTGGATCAACTGGATAACTCAAGCTAACCAAAATCATCCCAAACTTCCCACCCCATACCCTATTACCACTGCCAATTACCTGTGGTTTCATTTACTCTAAACCTGTGATTCCTCTGAATTATTTTCATTTTAAAGAAATTGTATTTGTTAAATATGTACTACAAACTTAGTAGT

**Supplementary Table 3.** The primer sequences used for RT-PCR analysis

| **Gene name** | **Forward Primer Sequence (5’-3’)** | **Reverse Primer Sequence (5’-3’)** |
| --- | --- | --- |
| AKT1 | TGGACTACCTGCACTCGGAGAA | GTGCCGCAAAAGGTCTTCATGG |
| AKT2 | CATCCTCATGGAAGAGATCCGC | GAGGAAGAACCTGTGCTCCATG |
| AKT3 | CGGAAAGATTGTGTACCGTGATC | CTTCATGGTGGCTGCATCTGTG |
| BAD | CCCAGAGTTTGAGCCGAGTG | CCCATCCCTTCGTCGTCCT |
| CAS9 | GTTTGAGGACCTTCGACCAGCT | CAACGTACCAGGAGCCACTCTT |
| DEC2 | CTGGGACATCTGGAGAAAGCTG | AGTGGAACGCATCCAAGTCCGA |
| ERK1 | TGGCAAGCACTACCTGGATCAG | GCAGAGACTGTAGGTAGTTTCGG |
| ERK2 | ACACCAACCTCTCGTACATCGG | TGGCAGTAGGTCTGGTGCTCAA |
| MEK1 | GGTGTTCAAGGTCTCCCACAAG | CCACGATGTACGGAGAGTTGCA |
| MEK2 | AGGTCCTGCACGAATGCAA | CGTCCATGTGTTCCATGCAA |
| MEK3 | CTTGGTGACCATCTCAGAACTGG | CTTCTGCTCCTGTGAGTTCACG |
| PLCG1 | CATCACGCACTACCAGCAGGTG | GACGCGCATTAGCATGTGCTCA |
| PLCG2 | AGCGACTCCTATGCCATCACCT | AACTGACGAGCTCCACCAGACT |
| PKC | GCCTATGGCGTCCTGTTGTATG | GAAACAGCCTCCTTGGACAAGG |
| PI3KA | GAAGCACCTGAATAGGCAAGTCG | GAGCATCCATGAAATCTGGTCGC |
| PI3KB | GGTAATCGGAGGATAGGGCAGT | CGGCAGTATGCTTCAAGGATGAC |
| PI3KG | GAGAGCTTGGAGGACGATGATG | CCACGCTTCAGCAGAAATCTGG |
| SPHK1 | AGAGTGGGTTCCAAGACACCT | GGGTGCAGCAAACATCTCAC |
| SPHK2 | GAGGAAGCTGTGAAGATGCCTG | GAGCAGTTGAGCAACAGGTCGA |
| SRC | CTGCTTTGGCGAGGTGTGGATG | CCACAGCATACAACTGCACCAG |
| VARP | CTTCAGCACCTTCCAGATCACAG | AGGAACAGGCTTCCTTCTCCAG |
| ACTB | CATGTACGTTGCTATCCAGGC | CTCCTTAATGTCACGCACGAT |

**Supplementary Table 4.** The antibody used for western blot analysis

| **Gene name** | **Manufacturer** | **Lot. No** | **Source** | **Dilution** |
| --- | --- | --- | --- | --- |
| AKT (pan) | Cell Signaling Technology | 4691T | Rabbit | 1:1000 |
| Phospho-AKT(Thr308) | Cell Signaling Technology | 9275S | Rabbit | 1:1000 |
| Caspase 9 | Cell Signaling Technology | 9508T | Mouse | 1:1000 |
| Cleaved caspase 9 | Cell Signaling Technology | 9509T | Rabbit | 1:1000 |
| P44/42 MAPK(ERK1/2) | Cell Signaling Technology | 4695T | Rabbit | 1:1000 |
| P-P44/42 MAPK | Cell Signaling Technology | 4370T | Rabbit | 1:1000 |
| VEGFR2 | SANTA CRUZ | Sc-6251 | Mouse | 1:1000 |
| Phospho-VEGFR2(Tyr1175) | Affinity | AF4426 | Rabbit | 1:1000 |
| Phospho-VEGFR2(Tyr951) | Affinity | AF3281 | Rabbit | 1:1000 |
| BHLHB3(DEC2) | Affinity | AF0442 | Rabbit | 1:1000 |
| SH2D2A | Affinity | DF4497 | Rabbit | 1:1000 |
| PI3K | Affinity | AF6241 | Rabbit | 1:1000 |
| Phospho-PI3K | Affinity | AF3241 | Rabbit | 1:1000 |
| Caspase 3 | Affinity | AF6311 | Rabbit | 1:1000 |
| Cleaved caspase 3 | Affinity | BF0711 | Rabbit | 1:1000 |
| PLCG1 | Affinity | AF6210 | Rabbit | 1:1000 |
| Phospho-PLCG1 | Affinity | AF3210 | Rabbit | 1:1000 |
| PKC | Affinity | AF6197 | Rabbit | 1:1000 |
| SPHK1 | Affinity | DF6005 | Rabbit | 1:1000 |
| VEGFC | Affinity | DF7011 | Rabbit | 1:1000 |
| HIF1α | Affinity | BF8002 | Mouse | 1:1000 |
| β-Actin | Affinity | T0022 | Rabbit | 1:1000 |
